# Structure and Sequence Determinants Governing the Interactions of RNAs with Influenza A Virus Non-Structural Protein NS1

**DOI:** 10.3390/v12090947

**Published:** 2020-08-27

**Authors:** Alan Wacquiez, Franck Coste, Emmanuel Kut, Virginie Gaudon, Sascha Trapp, Bertrand Castaing, Daniel Marc

**Affiliations:** 1 Equipe 3IMo, UMR1282 Infectiologie et Santé Publique, INRAE, F-37380 Nouzilly, France; alanwacquiez@gmail.com (A.W.); emmanuel.kut@inrae.fr (E.K.); sascha.trapp@inrae.fr (S.T.); 2 UMR1282 Infectiologie et Santé Publique, Université de Tours, F-37000 Tours, France; 3 Centre de Biophysique Moléculaire, UPR4301 CNRS, rue Charles Sadron, CEDEX 02, 45071 Orléans, France; franck.coste@cnrs-orleans.fr (F.C.); virginie.gaudon@cnrs-orleans.fr (V.G.)

**Keywords:** influenza A virus, non-structural NS1, RNA, RNA-protein interaction, 3D structure

## Abstract

The non-structural protein NS1 of influenza A viruses is an RNA-binding protein of which its activities in the infected cell contribute to the success of the viral cycle, notably through interferon antagonism. We have previously shown that NS1 strongly binds RNA aptamers harbouring virus-specific sequence motifs (Marc et al., Nucleic Acids Res. 41, 434–449). Here, we started out investigating the putative role of one particular virus-specific motif through the phenotypic characterization of mutant viruses that were genetically engineered from the parental strain WSN. Unexpectedly, our data did not evidence biological importance of the putative binding of NS1 to this specific motif (UGAUUGAAG) in the 3′-untranslated region of its own mRNA. Next, we sought to identify specificity determinants in the NS1-RNA interaction through interaction assays in vitro with several RNA ligands and through solving by X-ray diffraction the 3D structure of several complexes associating NS1′s RBD with RNAs of various affinities. Our data show that the RBD binds the GUAAC motif within double-stranded RNA helices with an apparent specificity that may rely on the sequence-encoded ability of the RNA to bend its axis. On the other hand, we showed that the RBD binds to the virus-specific AGCAAAAG motif when it is exposed in the apical loop of a high-affinity RNA aptamer, probably through a distinct mode of interaction that still requires structural characterization. Our data are consistent with more than one mode of interaction of NS1′s RBD with RNAs, recognizing both structure and sequence determinants.

## 1. Introduction

With 3–5 million severe cases and an excess mortality of 290–650 thousand deaths per year [1,2], influenza viruses remain one of the major infectious threats to human health. In addition to this seasonal disease burden, the sporadic human cases of infection with avian influenza viruses (mainly H5N1 and H7N9) are a reminder of the pandemic potential of these emerging viruses if ever they acquired the ability to transmit between humans. Due to the limits of vaccination strategies targeting the mutation-prone influenza viruses, antiviral therapies are still a valid option, notably for the treatment of severe influenza-related respiratory infections, in both seasonal and pandemic influenza.

While the first generation of influenza antivirals, adamantanes, have become obsolete due to the acquired resistance of the currently circulating viruses [3], neither the neuraminidase inhibitors nor the recently approved viral-polymerase inhibitor baloxavir marboxil [4,5] are safe from the development of resistances [6]. As a consequence, there is a pressing need to enrich our assortment of antivirals, both to improve our preparedness against future pandemics and to design combination therapies that are less prone to the emergence of resistances.

Several viral proteins could be targeted by novel antiviral compounds that could be used alone or in combination. One of the most promising targets is the influenza virus non-structural protein 1 (NS1) [7]. Encoded by the smallest of the eight viral genome segments, NS1 is a multifunctional RNA-binding protein, which is considered the main weapon of the virus to antagonize the innate immune response of the host and to ensure viral replication in a hostile anti-viral environment [8]. This 230-residue protein is highly expressed in the infected cell, where it exhibits several pro-viral activities [9,10]. Its homodimeric RNA-binding domain (RBD, amino-acids 1–73) interacts with several RNAs, including viral mRNAs [11,12,13,14,15], while its Effector Domain (ED, amino-acids 80–202) is a non-obligate dimer that interacts with several cellular proteins. A linker region connecting the two structured domains [16,17] confers plasticity to the quaternary structure, while the C-terminal tail (amino-acids 202–230) has the properties of an intrinsically disordered peptide region [17]. While the effector domain is to some extent dispensable for the biological activities of NS1 [14,18], the RBD plays an essential role in several activities that require the interaction of NS1 with viral and non-viral RNAs. This is best illustrated by the total loss of pathogenicity conferred by the alanine substitution of Arg38 and Lys41, the two main residues involved in binding of RNAs by NS1 [19].

In agreement with its cellular protein partners that have been identified in systematic searches [20,21,22,23], NS1 inhibits several pathways involved in the interferon activation and antiviral state [24] and also blocks the processing and nucleo-cytoplasmic export of host mRNAs [21,25]. However, when performing its RNA-related biological activities, NS1 may be selective, either towards some virus-derived RNAs or towards some subset of host cell RNAs, depending on the presence of sequence or structure determinants. Indeed, a systematic crosslink-based search revealed that NS1 preferentially binds introns in a subset of mRNAs, including that of the retinoic-acid induced gene RIG-I [26]. On the other hand, the dsRNA-binding property of NS1 was shown to be required for its inhibition of helicase DHX30 in that it is mediated by a dsRNA that is bound by both proteins [27]. The sequence and structure elements that control the interaction or determine its biological consequences are for the most part unknown. In a previous work [13] we have shown that NS1 binds in vitro with high affinity to RNA aptamers harboring virus-specific RNA motifs. Two conspicuous virus-specific motifs were identified: AGCAAAAG, which is strictly conserved at the 5′end of the virus-derived positive-strand RNAs, and UGAUUGAAG, which is highly conserved in the 3′untranslated region (3′UTR) of NS1′s own mRNA. We also showed that NS1 strongly interacts with double-stranded (ds) RNAs containing the GUAAC motif.

In the present work, we first investigated the role of one particular putative NS1-binding site in the 3′UTR of its own mRNA through the phenotypic characterization of mutant viruses that were genetically engineered from the parental strain WSN. In a complementary approach, we explored the hypothesis that NS1 may recognize the virus-specific motifs or the GUAAC motif through specific modes of interaction that might be distinct from what was described for the published structures of NS1-dsRNA complexes [28,29]. To identify such a specific NS1-RNA interaction that could represent a promising target of novel antiviral drugs, we purified a large variety of RNA-binding domains (RBDs) that were representative of NS1′s sequence diversity. Through in vitro interaction assays, we analyzed the contribution of structure and sequence determinants in a series of aptamer-derived RNAs. Finally, we solved the crystal structure of several complexes, associating NS1′s RBD with dsRNAs of high and low affinity.

## 2. Materials and Methods

### 2.1. Rescue of Recombinant Influenza Viruses

Wild-type (WT) and NS mutants of virus A/WSN/1933 (H1N1) were generated by reverse genetics using the 12-plasmid reverse genetics system [30] kindly provided by Ervin Fodor (University of Oxford, Oxford, UK). Mutations in the NS segment-plasmid were introduced by site-directed mutagenesis using the QuikChange II site-directed mutagenesis kit (Agilent, Santa Clara, CA, USA) according to the manufacturer’s protocol. The recombinant viruses were rescued by reverse genetics as described previously [31]. The presence of the desired sequence alterations in the amplified viruses was verified by sequencing their NS gene segment using reverse transcription (RT)-PCR.

### 2.2. Cells, Infections, Virus Titration, and Multicycle Growth Kinetics

HEK293T cells were maintained in Dulbecco’s modified Eagle medium supplemented with 10% fetal calf serum, 2 mM L-glutamine, 100 IU/mL penicillin, and 100 mg/mL streptomycin. Madin-Darby canine kidney (MDCK) cells and A549 cells were grown in Eagle’s minimal essential medium (EMEM) supplemented with 7.5% fetal calf serum. For virus growth kinetics, subconfluent monolayers of MDCK in 75cm^2^-flasks were virus-infected at a multiplicity of infection of 0.001 PFU/cell. Following 1 h of adsorption at 37 °C, the cells were further incubated in serum-free DMEM containing 1 µg of TPCK-treated trypsin (Worthington Biochemicals, Lakewood, NJ, USA). Samples of supernatants that were taken at the indicated times were subsequently titrated by plaque assays on MDCK cells [32].

### 2.3. Immunodetection of NS1 and NP

Subconfluent monolayers of A549 cells in 24-well plates were virus-infected at a multiplicity of infection of one PFU/cell. At the indicated times, cells were lyzed in 100 µl of Laemmli buffer and an aliquot was used for electrophoretic separation and immunoblot detection using the NS1- and NP-specific polyclonal rabbit antisera (anti-NP PA5-32242, Thermo Fischer Scientific, Waltham, MA, USA) and the ECL-system detection (Advansta, San Jose, CA, USA).

### 2.4. Minigenome Assay

Eukaryotic expression vector pCIwt-NS1 encoding wt-NS1 was constructed by sub-cloning its coding sequence between the *Xho*I and *Not*I sites of the pCI plasmid (Promega, Madison, WI, USA). In order to prevent production of spliced mRNAs, splice-donor and splice-acceptor sites were both invalidated by point mutations [33]. Substitutions within NS1 (R38A-K41A) were introduced using the QuikChange II site-directed mutagenesis kit. Subconfluent HEK293T cells in 24-well plates (one technical triplicate for each condition) were transfected using the FuGENE (Promega, Madison, WI, USA) reagent with the expression vectors of the four viral polymerase subunits (PB1, PB2, PA, and NP) and the pPolI-NS-Renilla encoding the chimeric minigenome (consisting of the NS genome segment with NS1′s Open Reading Frame (ORF) replaced by the Renilla Luciferase ORF), along with the pCI-NS expression vector (or empty vector as a control). The pCMV-Firefly plasmid (Promega) was used as control for transfection efficiency. Twenty-four hours post-transfection, cells were lysed and the activities of the two luciferases were measured using the Dual-luciferase reporter assay system (Promega) and a GloMax-Multi microplate luminometer (Promega). The minireplicon-driven Renilla-luciferase activity was normalized with respect to the activity of the Firefly luciferase, which was used as a transfection control.

### 2.5. RNA Probes and Proteins

All RNA probes used in binding experiments were purchased from Eurogentec (Belgium). AWFC01 and ZKO* RNA probes used in crystal structure investigations were purchased from *Dharmacon* (Open Biosystem, Horizon Discovery, Waterbeach, UK) (Figure S1).

The nucleotide sequences of RBDs (aa 1-73) were either PCR-amplified with appropriate primers from the cDNAs of the corresponding viruses or purchased as synthetic genes (Eurogentec, Liege City, Belgium), then cloned into the *Nde*I-*BamH*I sites of the expression vector pET15b(+) DNA (Novagen, Merck, Germany). The R38A-K41A double substitution was introduced using the QuikChange kit (Agilent, Santa Clara, CA, USA). In addition, the full-length NS1 sequence of the avian H7N1 virus was purchased as a synthetic gene cloned into the *Spe*I-*BamH*I sites of expression vector pGEX-5X-1 DNA (Genscript, Piscataway, NJ, USA).

Recombinant proteins were overproduced in Rosetta2-(DE3) bacteria in auto-induction medium [34]. Bacteria were grown at 37 °C until reaching an OD600 value of 0.6, then 20 h at 18 °C with agitation (200 rpm). Bacteria were harvested by centrifugation and stored at −20 °C as a dry pellet in a 50 mL polypropylene tube.

Bacterial pellets were gently thawed on ice and resuspended in 45 mL of buffer containing 20 mM Hepes pH 7.6, 500 mM NaCl, and 5 mM Imidazole. The bacteria were lyzed by a 30 min-incubation at 30 °C with 0.75 mg/mL of lysozyme, followed by three freeze/thawing cycles and five 1 min sonication cycles (amplitude 0.4, five pulses per second). The lysate was clarified by a 80 min centrifugation (19,000× *g*, 4 °C). The cleared lysate was applied onto a metal affinity-chromatography column (HisTrap FF, GE Healthcare, Chicago, IL, USA). Untagged proteins were eliminated through successive washes with (i) 10 column-volumes (cv) of wash buffer (20 mM Hepes pH 7.6, 500 mM NaCl) containing 5 mM Imidazole, (ii) 10 cv of wash buffer containing 20 mM Imidazole, and (iii) 10 cv with (20 mM Hepes pH 7.6, 2 M NaCl). His-tagged proteins were eluted by slow application of 10 cv of wash buffer containing 200 mM Imidazole. Fractions containing recombinant proteins (as verified by SDS-PAGE) were loaded onto a POROS HS20-cation exchange chromatography column (Applied Biosystems, Foster City, CA, USA) and eluted by a linear NaCl gradient (20 mM Hepes pH 7.6, 1 M NaCl). Elution fractions containing the protein of interest were concentrated (Amicon^®^Ultra 10K, Millipore, Burlington, MA, USA) to a 10 mL final volume with successive cold-centrifugations (15–20 min at 5.000 g) before His-tag cleavage at 4 °C during 16 h in 20 mM Tris-HCl pH 8.4, 150 mM NaCl, and 2.5 mM CaCl2 by 1:100 molar ratio of thrombin (Millipore, Burlington, MA, USA). The resulting proteolysate was applied onto a benzamidin column (GE Healthcare) to eliminate thrombin. Recombinant proteins were applied onto a HisTrap FF (GE Healthcare) and eluted with 5 cv of 20 mM Hepes pH 7.6, 500 mM NaCl, and 5 mM Imidazole. Untagged proteins were separated by size-exclusion chromatography onto a Superdex 75 column (GE Healthcare) equilibrated in 20 mM Hepes pH 7.6 and 500 mM NaCl. Fractions of the elution peak of the protein of interest were pooled and concentrated as described previously and stored at −80 °C.

Bacterial pellets containing the fusion proteins GST-RBD and GST-NS1 (H7N1) were resuspended in 45 mL of PBS containing 1% Triton X-100 and 1 mM PMSF and the bacteria were lyzed as described above. The cleared lysate was applied onto a glutathione-affinity chromatography column (GSTrap FF, GE Healthcare). Unbounded proteins were eliminated by washing with 10 cv of PBS. The fusion protein was eluted with 10 cv of 50 mM Tris pH 8 and 10 mM reductive form of Glutathione. Elution fractions containing GST-fusion proteins (as checked by SDS-PAGE) were pooled and concentrated as described previously. The concentrated GSTrap elution fraction was then loaded onto a size-exclusion chromatography column (Superdex S200 or S75 for GST-NS1 and GST-RBD, respectively, GE Healthcare) equilibrated in 25 mM Tris HCl pH 7.6, 5% Glycerol, 1 M NaCl, 0.1 mM TCEP. The elution fractions containing the fusion protein were concentrated as described previously and stored at −80 °C. The homogeneity of all purified proteins was assessed by SDS-PAGE and by MALDI-TOF mass spectrometry.

### 2.6. Electrophoretic Mobility Shift Assays (EMSA)

5′-[^32^P]-labelled RNA probe (0.1 nM) was incubated at 4 °C for 30 min with indicated amounts of protein in a 20 µL-reaction mixture (10 mM Tris-HCl, 1 mM EDTA, 0.1% bovine serum albumin, 10% glycerol, 50 mM NaCl, pH 8, 4 µM of yeast tRNA (*Sigma-Aldrich*)). RNA species were separated by electrophoresis (14V/cm at 4 °C) through a non-denaturing 10% polyacrylamide gel (29:1 acrylamide:bisacrylamide) in TBE buffer. Gels were subsequently dried and exposed for autoradiography. RNA species (free and bound to protein) were quantified using the Typhoon-FLA 9500 imager (GE Healthcare) and *ImageQuant* software, version TL v.8.1. Triplicate EMSA titration experiments were used to extract the dissociation constant K_D_ [35]. The binding curves were fitted using a non-linear regression logistics function (Hill’s equation, Y = [A2 + (A1−A2)]/[1 + (x/x_0_) p] with software Origins, version 9.0.0 (OriginLab, Northampton, MA, USA).

For competition experiments with unlabeled RNA competitors, radiolabeled AWFC01 (0.1 nM) was first incubated for 30 min with a limiting concentration of the indicated RBD (close to its K_D_ value). Subsequently, increasing concentrations of competitor were added and the mixture was incubated for an additional 30 min at 4 °C. The reaction mixtures were then separated by EMSA. Triplicate EMSA competition experiments were used to extract the apparent half-maximal effective concentration (EC50app) for the dissociation of the preformed AWFC01/RBD complex. After EMSA quantification, EC50app was determined by fitting the dose-response curves using the same equation as above.

### 2.7. X-ray 3D Structure Determination

Prior to crystallization, H7N1 NS1-RBD protein (wtH7N1-RBD) was concentrated to 10 mg/mL using Amicon 10 K ultrafiltration devices (Millipore) in last purification buffer. Initial crystallization screening was performed with a Mosquito liquid handler (STP Labtech Ltd., Royston, UK) using Morpheus, JCSG+, Wizard Classic, and Wizard Cryo kits from Molecular Dimensions Ltd. and the AmS04 kit from Qiagen. Diffraction-quality crystals appeared in Morpheus condition 2.5 (100 mM Hepes/MOPS pH 7.5, 8% ethylene glycol, and 30% P550MME-PEG20K) after few days and were flash-frozen directly into liquid nitrogen. Then, 100 K X-ray diffraction data were collected at PROXIMA 2 beamline (SOLEIL, Paris, France) and processed using XDS [36] and AIMLESS [37]. The 3D structure was determined at 1.93Å resolution by molecular replacement with Phaser [38] of the Phenix suite [39] and using PDBid 1AIL as a search model. The atomic model was refined using phenix.refine and manually improved using COOT [40]. Concerning the structures of H7N1-RBD/RNA complexes, double strand RNA (dsRNA) were prepared in 10 mM Tris, 1 mM EDTA pH 8.0, and 500 mM NaCl by mixing the two complementary single strand RNA synthesized and purified at Dharmacon, heating for 2 min at 90 °C and then being cooled down overnight to 4 °C. Freshly purified H7N1-RBD was mixed with dsRNA at a molar ratio of 1:1.1 and incubated for 30 min at 4 °C. Initial crystallization screening was realized as indicated for the H7N1-RBD protein alone. Crystals of the complex of wild type H7N1 NS1-RBD protein and AWFC01 dsRNA (wtH7N1-RBD/AWFC01) were obtained in Wizard Classic condition 2.26 (100 mM CHES pH 9.5 and 30% PEG400) and double mutant R38AK41A H7N1 NS1-RBD protein with AWFC01 dsRNA complex (aaH7N1-RBD/AWFC01 RNA) crystallized in Wizard Cryo condition E3 (100 mM sodium phosphate/citric acid pH 4.2, 200 mM ammonium sulfate and 40% ethylene glycol). After little optimization of AmSO4 kit condition 10, crystals of wild type H7N1 NS1-RBD protein bound to ZKO* dsRNA (wtH7N1-RBD/ZKO*) grew in 2 M ammonium sulfate and 200 mM ammonium nitrate. Then, 100 K X-ray diffraction data were collected at PROXIMA 2 beamline (SOLEIL, France) and processed using XDS [36] AIMLESS [37]. The 3D structures of wtH7N1-RBD/AWFC01, aaH7N1-RBD/AWFC01, and wtH7N1-RBD/ZKO* were determined at 1.75Å, 1.90 Å, and 2.30 Å resolution, respectively, by molecular replacement with Phaser [38] of the Phenix suite [39] and using PDBid 2ZKO as a search model. The atomic model was refined using phenix.refine and manually improved using COOT [40]. The 3D structures described here were deposited at the Protein Data Bank under access numbers 6SW8 (wtH7N1-RBD), 6SX0 (wtH7N1-RBD/AWFC01), 6SX2 (R38A-K41A-H7N1-RBD/AWFC01) and 6ZLC (wtH7N1-RBD/ZKO*). Data collections and refinement statistics are listed in Table S1. For the apo H7N1-RBD structure, residues 1 to 73 of the protein construct were visible in the electron density maps as well as three 1,2-ethanediol and one partial polyethylene glycol molecules. For the wtH7N1-RBD/AWFC01 complex, RBD residues E72 and T73 and RNA basepair G1:C19 could not be positioned by lack of electron density due to mobility, but six partial polyethylene glycol molecules and one CHES molecule could be located. Concerning the aaH7N1-RBD/AWFC01 complex, only residue T73 was missing and nine 1,2-ethanediol molecules were assigned. Lastly, RBD chain A residues −3 to 73 and RBD chain B residues 1 to 72, the whole double stranded RNA, as well as three sulfate ions and four nitrate ions were visible in electron density maps for the wtH7N1-RBD/ZKO* complex. Molecular graphics images were produced using UCSF Chimera [41]. RNA structure analysis was carried out using Curves+ [42]. RNA-Protein interactions were analyzed using the SNAP program of the 3DNA package [43].

## 3. Results

### 3.1. Biological Relevance of the UGAUUGAAG Motif

**Figure 1:**
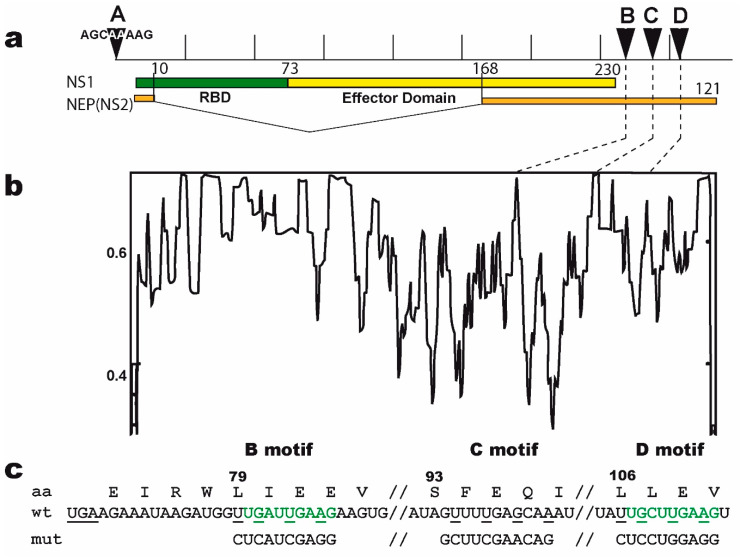
The non-structural (NS) segment and the mutated B, C, and D motifs. (**a**) The NS segment, showing the NS1 and NEP ORFs (NEP in orange; NS1 in green and yellow for the RBD and ED, respectively), with arrows indicating the positions of the A, B, C, and D motifs. Vertical bars = 100 nucleotides. (**b**) nucleotide conservation of NEP’s Open Reading Frame (ORF). The open reading frames of NEP from all human H1, H2, and H3 viruses were aligned and the nucleotide conservation (sliding window of 7 nucleotides) was plotted along the length of the ORF. The dotted lines highlight the relatively high conservation of motifs B, C, and D. (**c**) nucleotide sequence of the wt and mutated motifs, with the B and D motifs in green. The underlined UGA is the stop codon of NS1. The three sites are highlighted, with the mutated nucleotides underlined. Below are the three mutated motifs. The amino-acid sequence of NEP (shown above with L79, S93, and L106) remains unchanged. All possible combinations of mutants were rescued (i.e., single mutants B, C, and D; double mutants BC, BD, and CD; and the triple mutant BCD).

Our previous SELEX approach [13] identified two virus-specific RNA motifs in the NS1-binding aptamers, corresponding to short sequence motifs that are highly conserved in the virus-derived RNAs of positive polarity. The AGCAAAAG motif (here named motif A, Figure 1), which in the complementary RNA is part of the U12 sequence that makes up the viral polymerase promoter, is strictly conserved at the 5′end of all influenza A viruses complementary RNAs. At the same time, the UGAUUGAAG motif (here named motif B) is highly conserved in the 3′UTR of NS1′s mRNA (Figure 1). We first attempted to assess the biological relevance of the second motif, considering that for the first motif its well-established, prominent role in the viral cycle precluded a proper evaluation of its NS1-related role by mutagenesis of the viral genome. The UGAUUGAAG motif (or more generally UGRUUGAAG) is highly conserved in the 3′UTR of NS1′s mRNA, 14 nucleotides downstream of NS1′s stop codon. It corresponds to nucleotides 236–244 of Nuclear Export Protein (NEP)’s Open Reading Frame (ORF). In addition, a similar UGCUUGAAG motif (or less stringently, URYUUGAAG) is also present at the 3′end of the 3′UTR (nt 317–325 of NEP ORF). Strikingly, with about 2–4 occurrences in each NS-segment positive-strand RNA and a ratio of observed/expected frequency of 13.82, UUGAAG is the second most frequent six-letter word (AAUGGA is the first, at 14.93) in the nucleotide sequences of influenza A virus NS segments (~74,000 nucleotide sequences downloaded from the Influenza Virus Resource, available online: https://www.ncbi.nlm.nih.gov/genomes/FLU/Database/nph-select.cgi?go=database, accessed on 12 august 2020), representing about 63 million six-letter words).

On the basis of (i) the identification of the B motif by the SELEX approach, combined with (ii) the high conservation of its two copies (B and D) in the 3′UTR of NS1′s mRNA, we reasoned that NS1 could specifically interact with these motifs in its own mRNA and thereby regulate its translation. Accordingly, mutations in one of these motifs would be expected to alter both the synthesis of NS1 and the viral phenotype. We therefore introduced mutations in the NS-segment of virus A/WSN/33 and rescued the mutant viruses by reverse genetics. We modified either of the two UGRUUGAAG and URYUUGAAG motifs (i.e., B and D, respectively) in the 3′UTR of NS1, along with a third site, named C, that we used as a control. We were cautious as to not change the amino-acid sequence of NEP since motifs B, C, and D encompass the codons of amino-acids that belong to the α-helices C1 and C2 of the C-terminal part of NEP and are critical to its tertiary and quaternary structure as well as to its interactions with matrix protein M1 [44].

**Figure 2:**
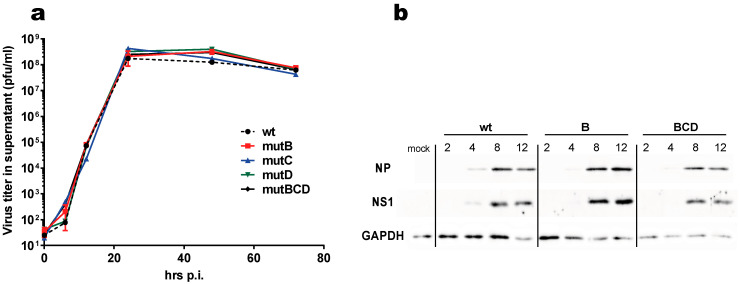
Multicycle growth curve of the three single mutants and of the triple mutant (**a**) MDCK cells that were infected with the indicated viruses (multiplicity of infection = 10^−3^ PFU/cell). Viral titers were measured in the supernatants that were collected at 6 h, 12 h, 24 h, 48 h, and 72 h post-infection. (**b**) Accumulation of NS1 and NP in virus-infected cells. A549 cells in a 24-well plate were infected (one PFU/cell), then lysed at the indicated times post-infection. Proteins in the lysates (2% of the 100 μL-lysate) were separated through SDS-PAGE, then transferred to a nylon membrane. NS1 and NP were revealed through immunoblot, using polyclonal rabbit antisera (anti-NP PA5-32242, *Thermo Fischer Scientific*).

All seven possible combinations of mutants were rescued, along with the wild-type virus. They all grew to similar titers on MDCK and their replication potential was similar to that of the wild-type virus, as shown by a multicycle growth assay in MDCK cells (Figure 2a). Their plaque phenotype was also undistinguishable from that of the wt virus (Figure S2). Further, we monitored the accumulation of viral proteins NS1 and NP in A549 cells that were infected with either the wt virus, single mutant B, or triple mutant BCD and noticed no difference between the three viruses regarding the accumulation of the two viral proteins (Figure 2b).

**Figure 3:**
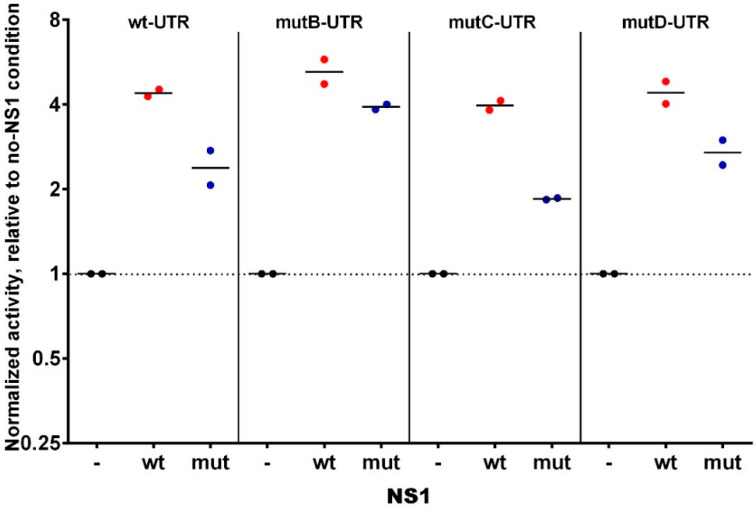
Modulation by NS1 of the activity of wt and mutated minigenomes HEK293T cells were transfected with the expression vectors of the polymerase subunits and the chimeric minigenome-expression plasmid, along with the NS1-expression vector (or empty vector as a control (-), or mutated-NS1-expression vector (mut)). Further, 24 h post-transfection, the activity of the Renilla Luciferase in the transfected cell lysate was measured and normalized relative to that of the Firefly luciferase activity. The normalized renilla activities were then expressed relative to that measured in empty-vector transfected cells (no NS1-condition). Each dot is the geometric mean of a technical triplicate in a given experiment (n = two independent experiments).

As an additional attempt to examine whether NS1 could somehow modulate the fate of the NS-viral genomic segment, we set up a “minireplicon” assay where NS1′s Open Reading Frame (ORF) in the NS-segment of WSN was replaced by that of the Renilla Luciferase. In this system based on the transfection of HEK293T cells, the normalized Renilla Luciferase signal reflects the replication, transcription, and translation of the chimeric “NS-Renilla”segment. We compared four such NS-Renilla segments, where the 3′UTR of NS1 corresponded to the wt sequence or to either of the three single-mutant sequences with the B, C, or D mutations. More specifically, we examined to what extent the transient co-expression of (wild-type) NS1 could impact the reporter signal (i.e., the replication, transcription, or translation). As shown in Figure 3, we repeatedly observed that transient co-expression of NS1 increased the minireplicon-signal by four- to six-fold compared to the signal measured with the same chimeric NS-Renilla segment in the absence of NS1. Similar foldchange ratios were observed with the four NS-Renilla segments, i.e., the foldchange was independent of the particular 3′UTR. Taken together, our data indicate firstly that NS1 dramatically increases the minireplicon efficiency, i.e., at least one of the three steps consisting of replication, transcription, or translation. Secondly, because this effect was observed regardless of the presence of the putative NS1-binding motifs in the 3′UTR of NS1 mRNA, it is unlikely that the putative binding, if any, of NS1 to the 3′UTR of its own mRNA has any relevance in the viral cycle.

### 3.2. Functional and/or Structural Insights into the NS1-RNA Interactions

#### 3.2.1. Minimal Structure of Selected Aptamers for Efficient Recognition by NS1

SELEX-identified aptamers DM01 and DM03 [13] have been selected for their interaction with a mixture of recombinant NS1 proteins representative of its two alleles A and B (from H5N1 and H7N1 viruses, respectively). These aptamers harbour one or two copies of the double-stranded motif GUAAC, along with a virus-specific sequence motif that is present in the apical loop of the long double-stranded hairpin, i.e., the AGCAAAAG motif in DM01 and the UGAUUGAAG motif in DM03 (corresponding to motifs A and B, respectively, in Figure 1). In order to identify the minimal RNA determinants required for binding, we first sought to identify shorter aptamer derivatives that still kept a high affinity for the RBD. Towards that aim, we designed two medium-length synthetic RNAs, DM01-midi and DM03-midi, that were derived from the 80-nt RNA-aptamers DM01 and DM03, respectively, along with shorter RNAs (DM01-short and DM03-short, respectively), consisting of the terminal hairpin of these aptamers encompassing the virus-specific motif. Their ability to form stable complexes with RBD were assessed through an electrophoretic mobility shift assay (EMSA) with the purified allele B-RBD (H7N1) (Table 1 and Figure S3).

While the medium-size dsRNAs DM01-midi and DM03-midi kept their binding properties (Table 1 and see below), the short RNAs DM01-short and DM03-short, which lacked the terminal GUAAC motif close to the free blunt end of their dsRNA structure, interacted poorly with the RBD. RNA-protein complexes with a shifted mobility were observed only at very high concentrations of the RBD (>2 µM), indicating a very low affinity (Figure S2). The two very short single-stranded RNAs consisting of only the 8- or 9-nt virus-specific sequence motifs (motifs A and B in DM01 and DM03, respectively) yielded no observable complex, even at the highest concentration of the RBD. Taken together, these data suggest that the duplex part of the hairpin RNA must extend at least over about 20 base pairs and must contain at least one GUAAC/CAUUG motif close to the blunt end of the dsRNA. Further, at least under the stringent binding conditions that we used, none of the virus-specific motifs that were identified by the SELEX approach showed a measurable, nanomolar-range affinity for the RBD in their isolated form.

#### 3.2.2. Major Contribution of the RBD in the Recognition of DM01-Midi and DM03-Midi

**Figure 4:**
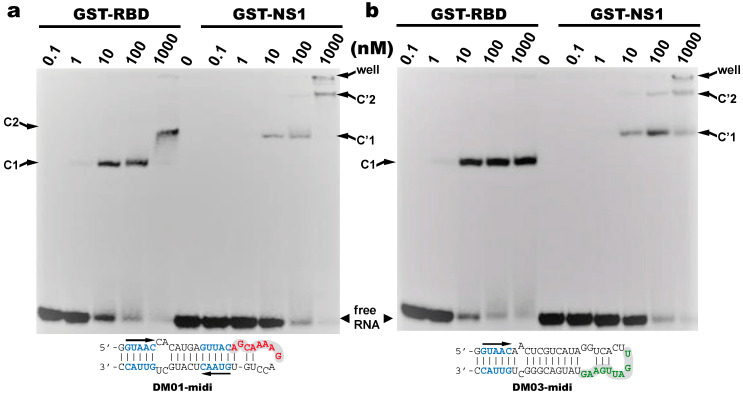
Binding of the RBD and full-length NS1 to DM01-midi and DM03-midi Radiolabeled DM01-midi (**a**) or DM03-midi (**b**) were incubated with increasing concentrations of GST-RBD or GST-NS1 full-length (H7N1). Free-dsRNAs and complexes were separated as described in Materials and Methods. C1 and C2 indicate the RBD:RNA complexes of 1:1 and 2:1 stoechiometry, respectively, while similarly, C’1 and C’2 indicate the NS1:RNA complexes of 1:1 and 2:1 stoechiometry.

We next assessed the interaction of DM01-midi and DM03-midi with the full-length protein NS1 and we chose to perform these experiments with the allele B NS1 from the avian H7N1 virus. Because the mature full-length protein was observed to precipitate even at moderate concentrations, these assays were performed with the fusion protein GST-NS1 or with GST-RBD as a control. Up to 100 nM of protein GST-RBD was observed to quantitatively titrate the two RNAs in a C1 complex, which likely corresponds to the binding of one GST-RBD molecule per RNA molecule (Figure 4). With DM01-midi at 1000 nM of GST-RBD, C1 was replaced by a second complex C2 (Figure 4a), likely of 2:1 stoichiometry, while under the same conditions, no additional complex was observed with DM03-midi (Figure 4b). This suggests that DM01-midi contains an additional determinant (not present in DM03-midi) that is recognized by a second GST-RBD, dependent on the binding of the first molecule. When GST-RBD was replaced by GST-NS1 in similar binding experiments, we observed that both RNAs formed a C’1 complex. In this case, however, not only for DM01-midi but also for DM03-midi, the C’1 complex was itself partially or completely replaced by a C2′ complex in the presence of higher concentrations of GST-NS1. This indicates that the formation of the low-mobility complexes (C2 and/or C’2), likely of 2:1 stoechiometry, required the presence of NS1′s effector domain for DM03-midi, while the RBD alone was sufficient in the case of DM01-midi.

**Figure 5:**
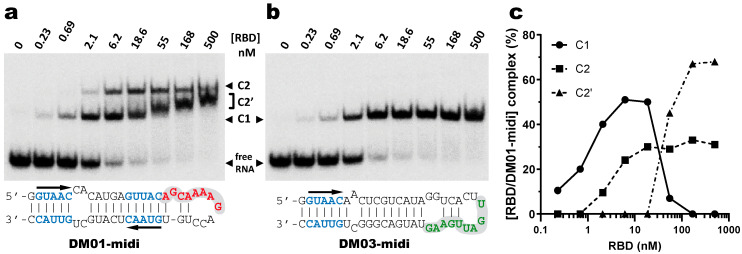
Interaction of DM01-midi and DM03-midi with the native H7N1-RBD Indicated radiolabeled RNA probes were incubated with increasing concentrations of RBD and the resulting reaction mixtures were analyzed by EMSA as described in Materials and Methods. (**a**,**b**) Representative gel autoradiographs obtained with DM01-midi and DM03-midi, respectively. (**c**) The relative intensities of the bands corresponding to the C1, C2, and C2′ complexes formed with DM01-midi were quantified and plotted as a function of the RBD concentration.

GST is known to be able to dimerize [45] and this property could favor the binding of several GST-NS1 molecules to the DM01-midi and DM03-midi RNAs. We therefore repeated our EMSAs using the native, untagged H7N1-RBD. The two RNAs were almost completely titrated by low nanomolar amounts of RBD (Figure 5). As was observed with GST-RBD, untagged RBD formed a unique C1 complex with DM03-midi, while at least two discrete complexes C1 and C2 were formed with DM01-midi. It is likely that the C1 complex observed with the two RNA probes corresponds to a 1:1 complex, associating one RBD dimer and one RNA molecule. The additional C2 complex that was observed only with DM01-midi was formed as soon as the RBD concentration reached 2 nM, even before the free RNA probe was completely titrated in C1. The quantification of EMSA suggests that C2 results from the facilitated binding of a second RBD to the 1:1 RBD:RNA C1 complex (Figure 5c). Above 20 nM of RBD, the apparent abundance of C2 remained relatively constant (around 30%), while C1 was progressively replaced by a C2′ complex, of which its diffuse and intermediate electrophoretic mobility gradually shifted towards that of the C2 complex. While its reduced electrophoretic mobility is indicative of a RBD:RNA stoichiometry above 1:1, the smeared appearance of C2′, along with the fact its position (its relative shift) is concentration-dependent, suggests that the C2′ and C1 complexes are in dynamic equilibrium during electrophoresis, with their relative amounts being reflected by the position of C2′. Taken together, these data suggest that as soon as the RBD concentration reaches 2 nM, a second RBD molecule binds to the preformed C1 to yield two distinct complexes of 2:1 RBD:RNA stoechiometry. Among these complexes, about 30% consist of the stable C2 complex, while the unstable one C2′ is in dynamic equilibrium with the 1:1 complex C1.

#### 3.2.3. Contribution of the AGCAAAAG and GUAAC Motifs to the Interaction

**Figure 6:**
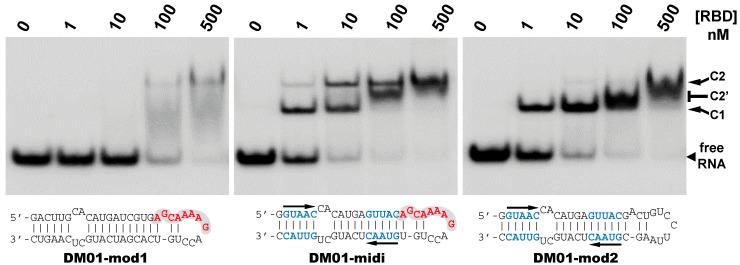
Comparative binding of RBD to DM01-midi, DM01-mod1, and DM01-mod2. The indicated radiolabeled RNA probes were incubated with increasing concentrations of H7N1-RBD and the incubation mixtures were analyzed by EMSA as described in Materials and Methods.

The different behaviors of the RBD towards DM01-midi and DM03-midi suggest that while both RNAs harbor a high affinity binding site that allows the formation of the 1:1 RBD:RNA ratio complexes C1, DM01-midi harbors an additional determinant that uniquely allows the formation of complexes C2 and C2′. The two RNAs share the long double-stranded structure as well as the terminal GUAAC motif, which together could form the high-affinity binding site. In addition, DM01-midi harbors a second GUAAC motif, close to the apical loop that also contains the virus-specific motif A (AGCAAAAG). In order to investigate the role played by each of these features, we designed and assessed the binding of DM01-mod1 and DM01-mod2, two hairpin RNAs derived from DM01-midi (Figure 6). DM01-mod1 conserved the virus-specific A motif while its dsRNA structure no longer contained the two GUAAC motifs. Conversely, DM01-mod2 kept the two GUAAC motifs but its A motif was replaced by an unrelated sequence.

RBD formed C2-like complexes with DM01-mod1, which harbors the AGCAAAAG motif, albeit only at high concentrations, while there was no discrete band corresponding to the C1 complex. Instead of C1, we observed a large smear (Figure 6). This suggests that RBD forms, with DM01-mod1 which harbors the AGCAAAAG motif, unstable C1-like complexes that dissociate during electrophoresis. Very differently, with DM01-mod2, which harbors the two GUAAC motifs, RBD at low concentrations (1–10 nM) formed C1 complexes, while at the 100 and 500 nM concentrations, unstable C2-like complexes of reduced mobility were observed. This last observation suggests that the presence of a loop of which its 2D structure is close to that present in DM01-midi and DM01-mod1 could facilitate the fixation of a second molecule on the preformed complex C1. However, because it lacks the viral sequence AGCAAAAG, this loop cannot form a stable C2 complex in the presence of moderate concentrations of RBD. Additionally, higher concentrations of the RBD induce the formation of unstable C2′ complexes that steadily dissociate during electrophoresis and yield the fuzzy band.

Noteworthily, the ability to form a C2 complex with the DM01-midi probe was shared by all the NS1 RBD proteins that we tested in this study, whilst only the C1 complex was observed with DM03-midi (Figure S4). The distinct electrophoretic mobility of the complexes are consistent with the divergent isoelectric points (pI) of the RBDs: for instance, the complexes made with the H5N1-RBD (pI 6.11) are more acidic and therefore less shifted than those formed with the H7N1-RBD (pI = 8.00). As expected, the double substitution R38A/K41A, either in the H7N1 or H5N1-origin RBD, abolished their binding to both RNAs.

Consistent with all the observations, we can propose the following scenario for the binding of RBD to DM01-midi. At subnanomolar RBD concentrations, RNA probes containing at least one GUAAC/GUUAC motif in their double stranded part are stably recognized by a first RBD molecule to form the C1 complex. In the 1–10 nM concentration range, a second RBD molecule binds to the preformed C1 complex in a cooperative-like manner that likely involves the recognition of the viral sequence (2:1 RBD:RNA ratio complex C2). Thus, the formation of a stable C2 complex intrinsically depends on a stable C1 complex and is favored by the presence of the viral sequence AGCAAAAG in the apical loop. The cooperative-like interaction with the AGCAAAAG motif is a property shared by all NS1 RBDs.

#### 3.2.4. AWFC01, a High-Affinity dsRNA Containing Two GUAAC Motifs

Our observations are consistent with the strong and preferential binding of the RBD to double-stranded RNAs harboring at least one copy of the double-stranded motif GUAAC. In order to establish the importance of this motif and of its location, we designed a series of short hairpin RNAs harboring either zero, one, or two copies of the GUAAC duplex. In order to avoid the binding of a second RBD molecule to the 1:1 complex of RBD with RNA, all RNA probes were designed with a GAGA apical loop that is known to stabilize the double stranded structure of RNA hairpins [46].

**Figure 7:**
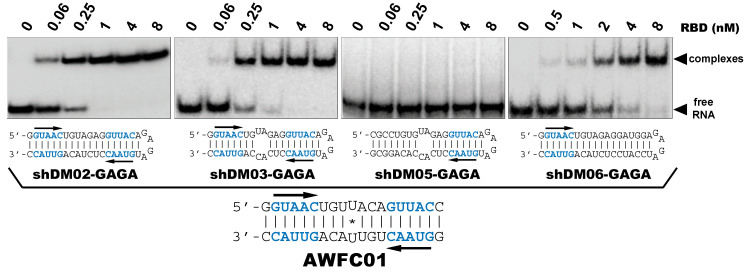
Importance of the number and position of the GUAAC motifs. The indicated radiolabeled RNAs were incubated with increasing concentrations of the H7N1-RBD, then analyzed by EMSA as described in Materials and Methods. Bottom: secondary structure of AWFC01, with its central U*U Hoogsteen base pair. The double-stranded GUAAC motif (blue) is emphasized and oriented by the horizontal arrow.

Nanomolar concentrations of RBD formed a unique 1:1 RBD:RNA ratio complex (C1-like) with shDM02-GAGA and shDM03-GAGA, both harboring two GUAAC motifs (Figure 7). Of the two short RNAs that contained only one GUAAC motif, a strong interaction was observed only with shDM06-GAGA, which harbored this motif at the blunt end of the hairpin RNA. In contrast, shDM05-GAGA, which harbors the unique GUAAC motif in the immediate vicinity of the GAGA loop, was not recognized by RBD in the nanomolar concentration range. Altogether, these data show that the RBD binds the GUAAC motif provided that the latter is readily accessible, being positioned at the free blunt end of the dsRNA, while contrariwise, its proximity with the apical loop probably hinders its accessibility or unfavorably alters its shape. Based on this rationale, we designed a model double-stranded RNA made up by the annealing of two synthetic RNA molecules of the same sequence. This synthetic palindromic dsRNA, named AWFC01 (Figure 7), harbors one GUAAC motif at each of its blunt ends and is perfectly symmetric thanks to a central uracil nucleotide that stabilizes the RNA duplex by forming a non-canonical U*U Hoogsteen base pair [47].

**Figure 8:**
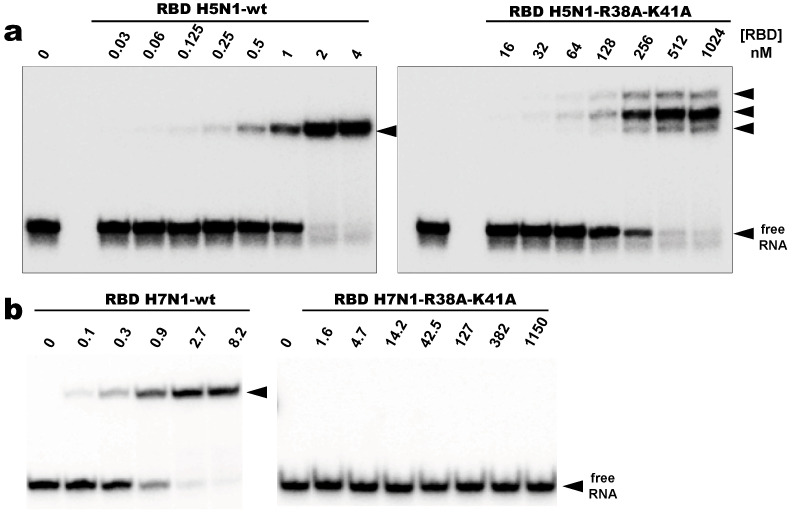
Binding of RBD to AWFC01 (**a**,**b**) Comparative EMSA titration experiments of AWFC01 by the wild type (wt) and the double mutant R38A-K41A RBD from H5N1 and H7N1, respectively. Radiolabeled AWFC01 was incubated in standard conditions with increasing concentrations of wild type or double mutant RBD in standard conditions and analyzed by EMSA. Representative gel autoradiographs are shown.

As expected, all wild type RBDs tested in this study recognized AWFC01 and formed a stable C1-like complex in a nanomolar range of protein concentrations (Figure 8a,b for H5N1- and H7N1-RBD, respectively). The dissociation constants extracted from EMSA titration experiments (Figure S5 and Table 2), with K_D_ values between 0.83 and 3.7 nM, place AWFC01 as the NS1 best dsRNA ligand described to date.

Surprisingly, the negative impacts of the double substitution (R38A-K41A) were very different for the H5N1 and H7N1 RBDs. Under the stringent binding conditions that we used, the mutant H7N1-RBD was unable to form a stable complex with AWFC01 (Figure 8b, right panel). At a ~50 µM RBD concentration, 50% of the RNA probe was titrated in unstable complexes that yielded a large smear by EMSA (data not shown). On the other hand, while the double substitution of the H5N1-RBD also dramatically reduced its affinity for RNA, a 400 nM concentration of protein was sufficient to titrate 50% of AWFC01 in several distinct complexes (Figure 8a, right panel). These were even more shifted than that formed with the wild type RBD. Their reduced mobility cannot result solely from the different isoelectric points of the wt (pI 6.11) and mutated (pI 5.23) proteins and we surmise that the complexes made with the mutant RBD are less compact. Further, the existence of three distinct complexes could originate either from a structural heterogeneity of the mutant protein or from distinct modes of interaction.

We also compared AWFC01 to the dsRNAs that were evaluated above (i.e., shDM02-GAGA, shDM03-GAGA, shDM05-GAGA, shDM06-GAGA, DM01-midi, and DM03-midi) as regards their affinity for the RBD. We also included in this comparison the model dsRNA that we named “ZKO”, the palindromic RNA duplex that was used to solve the crystal structure (PDBid 2ZKO) of H1N1-RBD bound to RNA [28]. To that end, we set up “competition EMSAs”, where increasing amounts of the unlabeled competitor RNAs were added after the incubation of radiolabeled AWFC01 with the protein (H5N1 or H7N1 RBD). After quantification of the free and bound fractions of the labeled AWFC01, we calculated the apparent half-maximal Effective Concentration (EC50_app_), expressed as the concentration of competitor required to shift the fraction of bound AWFC01 from 50% to 25%. As indicated by its very low EC50_app_ values (Figure S6 and Table 3), AWFC01 is among the best ligands for the two RBDs that we used. Although three of our dsRNAs also showed similarly low EC50_app_ values (shDM03, shDM02, and shDM06), they do not have the high symmetry of AWFC01. As for ZKO, its high EC50 value suggests a far lower affinity for the RBD.

### 3.3. Structural Basis of NS1′s Interaction with Model dsRNA AWFC01

In spite of the fact that AWFC01 harbored neither of the virus-specific motifs, both its symmetry and its high affinity for the RBDs prompted us to choose it for our subsequent structural characterization. While essentially all of the RBDs that we purified yielded high quality crystals when crystallized alone, we chose to solve the structure of the avian H7N1 RBD alone and in complex with AWFC01. In parallel and for the sake of comparison, we also crystallized and solved the structure of two other complexes: AWFC01, with the mutant H7N1 RBD harboring the double substitution R38A-K41A and the slightly modified ZKO*-RNA, with the wt RBD. Table S1 (Suppl. Data) summarizes the X-ray data collection and refinement statistics.

**Figure 9:**
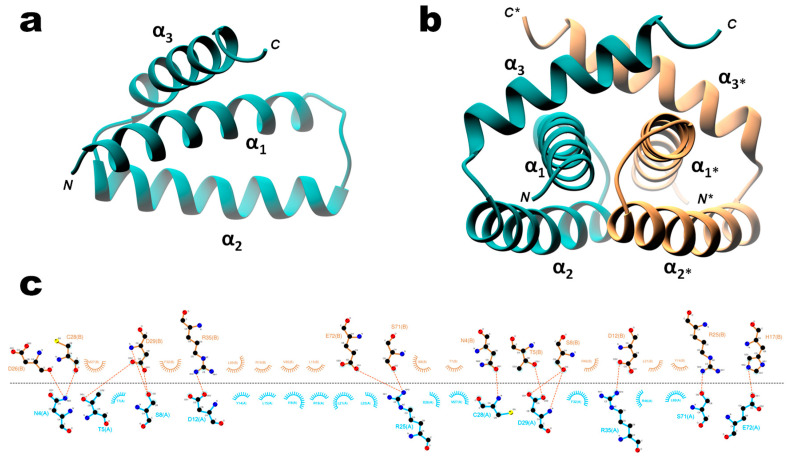
3D X-ray structure of wild type H7N1 NS1 RBD Ribbon representation of H7N1 NS1 RBD monomer (**a**) and homodimer (**b**), with chain A in dark cyan; chain B in salmon. (**c**) Ligplot representation of the dimer interfacing residues (with chains A and B colored as in b, and atoms colored in black, red and blue for carbon, oxygen and nitrogen atoms, respectively).

The X-ray structure of recombinant wild type H7N1 NS1 RBD (residues 1–73) was solved to 1.93 Å resolution. While it is the first structure that is representative of the RBD of allele B-NS1, its organization is identical to that of the other RBD structures available, forming a symmetric C2 homodimer shaped by three interlocking α-helices from each monomer. Helices α1 (residues S3-M24) and α2 (A30 to L50) are oriented in an antiparallel fashion, whereas helix α3 (L54-K70) sits perpendicular to helix α1 (Figure 9a). The dimerization interface, with an area of 1189Å2, involves 43% of the monomer residues, which are engaged in 15 H-bonds, including 4 salt bridges (PISA server). Superimposition of all available models shows that the six helices are positioned the same way (Figure S7a) and that only small structural divergences occur at the ends of the polypeptide chains. Most of the interfacing residues are located in helices α1 and α3 and in the turn between α1 and α2 (Figure S7b). Three highly conserved residues of α2 helix (F32, R35, and R46) are involved in the stabilization of the dimer, with R35 being engaged in a salt bridge with D12. Invariant residues R35, R37, R38, and K41, which are involved in RNA binding, adopt different conformations when comparing all the apo RBD homodimers (Figure S8a). Nonetheless (as seen in Figure 9b), the RNA binding interface formed by helices α2 and α2* of the homodimer seems to be well preserved on H7N1. This 3D structure also shows several ligands bound to the RBD, originating from the crystallization buffers. Three 1,2-ethanediol and one partial polyethylene glycol molecules have been assigned in a groove formed by helices α3 and α3*. Interestingly, another partial polyethylene glycol molecule is located in a nonpolar pocket found underneath the RNA binding channel at the RBD molecular two-fold axis (between helices α2 and α2*), making an H-bond with the side chain of R46(A). This arginine residue was also found to contact a malonate ion and a succinic acid molecule in the structure of H1N1 (PDB 3M8T) and H5N1 RBD (3F5T, [48]), respectively.

**Figure 10:**
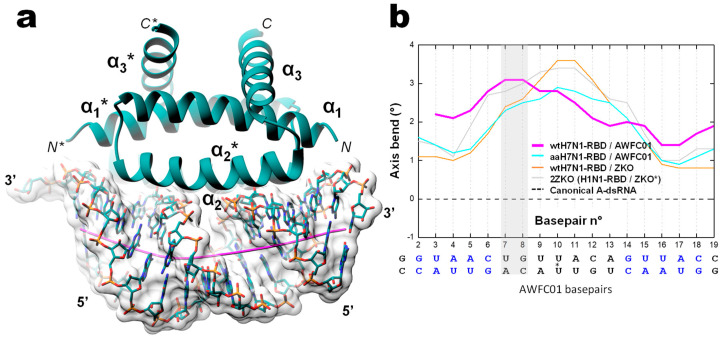
Overall structure of the complex between the wild type H7N1 NS1 RBD protein and the AWFC01 RNA (**a**) 3D structure of the wtH7N1-RBD/AWFC01 complex. The dsRNA axis calculated using Curves+ is represented as an orchid stick. (**b**) Plot of dsRNA axis bend versus base pair, calculated for wtH7N1-RBD/AWFC01 (pink), aaH7N1-RBD/AWFC01 (cyan), wtH7N1-RBD/zko* (orange), and 2ZKO (grey) complexes, relative to the canonical A-form dsRNA (dashed line). The secondary structure of AWFC01 is depicted below.

To get insight into the sequence-specific recognition of the double stranded RNA by the RBD, we solved, at 1.75 Å resolution, the crystal structure of the complex associating the wild type H7N1-RBD to the AWFC01 duplex (wtH7N1-RDB/AWFC01, Figure 10). The overall structure of the RBD in the complex is identical to that found in the apo form, with minor side chain rearrangements at the binding interface of helix α2 residues R37, R38, and K41 (Figure S7). In RBD chain A, the sidechain of R38 adopted two alternative conformations that were clearly distinguished in electron density maps. In the first conformation (“a” in Table 4), R38 makes two hydrogen bonds with the side chain of D39(A) and a third hydrogen bond with one strand of the RNA, between atoms R38(A).N(η2) and U7(C).OP1. In the second conformation (“b” in Table 4), the side chain of R38 sits in the middle of the RNA minor groove; it interacts with its symmetrically related residue and simultaneously contacts the phosphate groups of U7 nucleotides from the two strands (U7(C).OP1 and U7(D).OP2 atoms) and the phosphate group (OP1 atom) of C6 nucleotide from chain D (Table 4, columns 2 and 3). In RBD chain B, R37 side chain also adopts two conformations: in the first one, it interacts only with D34, while in the second one, it sits closer to the RNA, making H-bonds with the O2′ atom of U7(D) and with D34. The H7N1 RBD sits almost parallel to the dsRNA helix axis and overlays the minor and the major grooves of the duplex. The assembly buries 1173.8 Å2 of the RNA solvent-accessible area (17.1% of the total) and 1156.7 Å2 for the RBD (14.1% of the total). In the complex, the RNA keeps its A-form conformation but a curvature of about 20° is observed, stretching along base pairs 8 and 9 at one end of the duplex (Figure 10b). As shown in Table 4, all direct interactions between the H7N1 RBD and AWFC01 occur via phosphate/amino acid and sugar/amino acid H-bonds and none through base recognition. The sequence-specificity of the H7N1 RBD for AWFC01, which we observed in the preceding biochemistry experiments, could not easily be explained by the 3D structure of the complex. Of note, like in the apo-structure, we also identified in the structure of the wtH7N1-RBD/AWFC01 complex a partial PEG molecule that almost completely filled the pocket found underneath the RNA binding channel.

In order to search for complementary clues for the apparent sequence specificity of the H7N1 RBD for AWFC01, we solved the 3D structures of two other complexes: (i) R38A-K41A double mutant H7N1 RDB with AWFC01 (aaH7N1-RBD/AWFC01) and (ii) wild type H7N1 RBD, with the slightly modified nonspecific RNA “ZKO*” (wtH7N1-RBD/ZKO*). The dsRNA that Cheng et al. used to solve their structure [28] was slightly modified by removing its terminal, unpaired nucleotides. While the two complexes crystallized in different conditions and space groups, noteworthy similarities were observed (Figures S8 and S9) with low rmsd values of 0.33 Å for the proteins and 0.71 Å for the RNA. When comparing by superimposition the structures of the three RBD-RNA complexes, subtle structural differences were identified, mainly for the RNA molecule. Relative to the wtH7N1-RBD/AWFC01 complex, the RNAs in complexes aaH7N1-RBD/AWFC01 and wtH7N1-RBD/ZKO* differ by rmsd values of 2.00 Å and 2.21 Å, respectively, whereas the difference is much smaller as regards the RBD (rmsd of 0.26 Å and 0.33 Å, respectively, Figure S10). We observed the same difference when comparing the wtH7N1-RBD/AWFC01 complex to the 2ZKO complex, with rmsd values of 2.10 Å for the RNA phosphodiester backbone atoms and 0.45 Å for the protein main chain atoms. Since the main differences occurred at the RNA level, we used CURVES+ [42] to dissect and compare the nucleic acid conformations in the different complexes (Figures S11 and S12). Surprisingly, this revealed that in the high affinity dsRNA (AWFC01), the RNA axis is bent in a non-symmetrical fashion, whereas in the two other complexes (aaH7N1-RBD/AWFC01 and wtH7N1-RBD/ZKO*), the RNA axis bend is distributed symmetrically from the centre of the duplex (Figure 10b). For AWFC01, axis bending is at a maximum between the seventh and eighth basepair at one end of the duplex, right after the GUAAC sequence. At this position, the minor groove is less wide and deep than that at the other side of the dsRNA, while the opposite is seen for the major groove. This peculiar asymmetry results from a corresponding asymmetry in the network of amino-acid/nucleotides hydrogen bonds. As highlighted in Table 4 (shaded cells), several hydrogen bonds in the wt-RBD/AWFC01 complex are distinctively asymmetric, compared to their equivalents in the other structures: for instance, the η1 nitrogen atom of R38(chain A) interacts with the OP2 atom of U7(chain D) and contrary to the expected symmetrical relation that is observed in the other structures, the η1 nitrogen atom of R38(chain B) also interacts with U7 of the same chain D, this time with the OP1 atom. A similar relation is observed with the η2 nitrogen of R38 and U7 of chain C. As a consequence, the seventh nucleotide of both chains of the dsRNA are pulled by these interactions, bending its axis and skewing its shape.

Closer inspection of the protein-RNA interactions did not allow us to reveal sequence-related features that would account for the specificity and high-affinity of the RBD-AWFC01 interaction. As shown in Table 4, essentially four amino-acids formed a network of hydrogen bonds with either the phosphate groups or the ribose of the RNA backbone, with no sequence-specific bonds involving the nucleobases. Apart from the peculiarities of the hydrogen bonds involving the three nitrogen atoms of R38 (discussed above), the H-bond network is symmetric and involves the oxygen atom of P31, the γ oxygen atom of T49, and the η1 nitrogen of R35, which consistently make hydrogen bonds with O2′ of nucleotide 6 (P31), O2′ and O4′ of nucleotide 4 (T49), and O2′ of nucleotide 6 (R35 N(η1)). Interestingly, according to molecular dynamics simulations [49], P31 at the N-terminus of alpha-helix 2 is at the centre of the most flexible region in the RBD.

## 4. Discussion

In this work, we set out to investigate the importance of three distinct sequence and structure motifs in the interaction of influenza A virus NS1 with RNAs. These motifs were previously identified through an in vitro SELEX approach in which two recombinant proteins NS1, representative of its two alleles, were used to iteratively select high-affinity synthetic RNAs [13]. They included the double-stranded motif of sequence GUAAC, along with two sequence motifs of undetermined structure that are strictly or highly conserved in influenza virus A-derived positive strand RNAs: AGCAAAAG, which is strictly conserved at the 5′-end of all complementary RNAs and UGAUUGAAG, which is highly conserved in the 3′-UTR of NS1 mRNA, fourteen nucleotides downstream of NS1′s stop codon.

We first investigated the biological relevance of one of the two virus-specific motifs in various steps of the virus cycle. We considered it unrealistic to attempt to rescue mutant viruses with a mutated AGCAAAAG motif since it would be impossible to disentangle the putative NS1-related impacts from the expected impacts any such mutation in the viral polymerase promoter would have on the viral cycle. We therefore limited our in vivo approach to the study of viruses harboring mutations in the conserved motif UGAUUGAAG within the 3′UTR region of NS1′s mRNA. In addition and for the sake of comparison, we also mutated two other highly conserved motifs in the 3′UTR region of NS1′s mRNA, including the very similar sequence UGCUUGAAG, which is present at the distal end of this long 3′UTR. These mutations did not change the peptide sequence of NEP. All three single-mutant WSN viruses harboring either of these three mutated motifs (mutB, mutC, and mutD) were readily rescued and their phenotype in cultured cells was undistinguishable from that of the wt-virus. Furthermore, viruses harboring all three mutated sites also exhibited a wild type phenotype, as assessed (i) by the kinetics of multicycle virus growth and (ii) by the kinetics of viral protein (NS1 and NP) accumulation in mutant virus-infected cells. We also attempted to evaluate whether NS1 could modulate the activity of the viral polymerase on a “minireplicon” consisting of the NS genome segment where NS1′s ORF was replaced by that of the Renilla luciferase and whether mutations in the B, C, or D motifs could alter the replication, transcription, or translation of this minigenome. Indeed, we consistently observed that NS1 dramatically increased the activity of the viral polymerase (4-fold to 6-fold increase). However, this polymerase-enhancing activity of NS1 was equally observed, with the minireplicons harboring either of the B, C, or D mutation, thus ruling out any major role of these motifs in this observed activity. Moreover, even when the minireplicon system was complemented with the R38A-K41A NS1 rather than with wt-NS1, the enhancing activity was still observed, albeit to a lower level. This suggests that the polymerase-enhancing activity does not rely solely on the RNA-binding ability of the RBD. Indeed, several studies had shown that NS1 interacts with the viral polymerase [50], most likely through a direct interaction of its RBD with the viral nucleoprotein [51]. It should be highlighted, however, that the read-out of the minireplicon system (i.e., the Renilla luciferase activity) results from at least three activities combined: (i) replication and (ii) transcription of the minigenome by the viral polymerase, along with (iii) translation of the viral-like mRNA. Therefore, it is likely that the observed effects of NS1 on that system combine the enhanced activity of the viral polymerase with the increased translation of the viral-like mRNA, which has been demonstrated by several studies [14,52,53]. Taken together, although our data confirm the important activity of NS1 in the viral cycle (notably in relation with the viral polymerase and the translation of viral mRNAs), they provide no evidence for our initial hypothesis that NS1′s binding to the 3′UTR of its own mRNA could be critical to the viral cycle.

In spite of the apparently negligible relevance of the putative interaction of NS1 with the B motif, we stress that we could not assess, in the context of the viral cycle, the biological relevance of its interaction with motif A, i.e., the AGCAAAAG motif at the 5′end of all virus-derived RNAs of positive-polarity. These two virus-specific motifs probably represent only a fraction of the putative NS1-binding motifs in viral or cellular RNAs and indeed, a systematic search showed that NS1 preferentially binds intronic sequence motifs in a subset of host cell mRNAs including that of the retinoic-acid induced gene RIG-I [26], resulting in decreased processing of pre-mRNAs and a reduced expression. This illustrates the view that the high concentration of NS1 in the infected cell [19], combined with its presence in several subcellular compartments, allows it to interact, with a broad range of affinities, with several RNAs that could represent the targets of its biological activities. We therefore chose to study in depth the determinants that allow a given RNA to interact with NS1, starting from the RNA aptamers that had been selected previously through the SELEX approach.

We first showed that the RBD preferentially interacted with unbranched dsRNA structures with a minimal length of about 20 base pairs. While we observed no high-affinity interaction of the RBD with short single-stranded RNAs consisting only of the two 8 nt- or 9 nt- virus-specific sequence motifs, our data show that the RBD, by itself, was able to interact with the AGCAAAAG motif with no obvious involvement of the effector domain. This sequence-specific interaction was observed to occur cooperatively on a preformed 1:1 RBD:RNA ratio complex, associating one RBD bound to the dsRNA structure of aptamer derivative DM01-midi. We hypothesize that this first interaction induces a structural change in the dsRNA that propagates to its apical loop, thereby facilitating the binding of a second RBD to this apical loop containing the AGCAAAAG motif. Such a behavior was not observed in the other aptamer derivate, but in that case, we observed that the effector domain was required for a cooperative-like behavior and we surmise that the ED specifically recognizes the second virus-specific motif, UGAUUGAAG, or contributes at its RBD recognition. The structural dynamic of full-length NS1, especially regarding the relative orientation of its two domains [16,17], could also be involved in this cooperative RNA binding between NS1 molecules and the RBD in the solution could exist in equilibrium between different conformations that are non-equivalent for the cooperative binding to RNA.

We then chose to focus on the interaction of NS1 with the double-stranded structure. Altogether, our experiments had shown that an optimal interaction with the RBD required the presence of the GUAAC motif positioned close to the free blunt end of the dsRNA. We especially addressed the question, what mechanism could allow the RBD to specifically recognize this sequence, given the non-specificity that is generally observed in dsRNA-binding proteins? Such a lack of specificity is notably observed in the B2 protein of Flock House virus [54], which like NS1, interacts with dsRNA through two antiparallel alpha-helices of the same sequence (PDB 2AZ2). Indeed, there is a notorious paradox in the sequence recognition within double-stranded RNAs since the nucleobases are not easily accessible [55,56] and most of the interactions involve either the phosphodiester backbone or the 2′-hydroxyl groups of the ribose moiety. In order to elucidate this point, we designed AWFC01, a model double-stranded RNA of perfect symmetry containing a central U*U Hoogsteen base pair. We showed the very high affinity of this model dsRNA for the RBD and determined the structure of three RBD-dsRNA complexes, including two with AWFC01. Unexpectedly, the high symmetry of both the secondary structure of AWFC01 and the 3D structure of the RBD did not result in a corresponding symmetry of the dsRNA 3D structure in the complex. Instead, we observed a pronounced asymmetry in the network of hydrogen bonds that links the dsRNA to the RBD, owing to the position of residue R38 in polypeptide chain A. This skewed network pulls the seventh nucleotide of the two RNA chains, thereby locally bending its axis and skewing the overall shape of the dsRNA. This probably constitutes an important determinant in the sequence-specific recognition of the dsRNA. The A-form helix of the dsRNA can undergo subtle conformation changes that alter its shape and it is believed that sequence specificity in dsRNAs often relies on recognition of shape [56,57], which in itself can be dependent on the underlying sequence of the dsRNA. We hypothesize that the affinity of the dsRNA-RBD interaction is dramatically increased by the altered shape of the RNA or more precisely, by the sequence-dependent predisposition to adopt this given shape.

Besides allowing us to propose an explanation to the sequence specificity within dsRNAs, our structure and the distinctive bending of the dsRNA axis suggests that the RBD interaction can indeed alter the conformation of the dsRNA (although we did not solve the structure of the RNA alone). This conformational change likely can propagate in RNAs with more complex structures and as discussed above, this could result in aptamer-derived DM01-midi in the cooperative binding to the virus-specific sequence in its apical loop, through a necessarily distinct binding mode. We stress that our study was performed with a bacterially-expressed NS1, while several studies have shown that NS1 can be modified post-translationally, notably by phosphorylation at several sites including serine 48 and threonine 49 in its RNA-binding interface [58,59,60,61]. It was also shown that phosphorylation of threonine 49 abolished the anti-interferon activity of NS1 and slightly reduced its RNA-binding activity [60]. Phosphorylation of Ser 48 and Thr 49 in the infected cell could therefore provide an optional switch that modulates the RNA-binding capacity of NS1, adding a layer of complexity in the dynamic network of NS1-RNA interactions in the infected cells.

While keeping in mind that the high-affinity ligands are not always optimal for the biological function [62], we acknowledge that our data do not provide direct evidence as to the biological relevance of these sequence-specific interactions of NS1 with RNAs and do not support the importance of the putative interaction of NS1 with its own mRNA. However, our structural and biochemical data, combined with the known abundance of NS1 in the infected cell, support the view that NS1, through its RBD, can interact by more than one single mode of binding and with a broad range of affinities, with several distinct RNAs in the infected cell. As we have hypothesized previously [13], NS1 could compete with spliceosomal protein U1C in its interaction with the U1-mRNA duplex at the splice donor site [63,64], owing to its preferential recognition of the GUAAC motif. Further, the specific recognition of the AGCAAAAG motif most likely plays an important role in the viral cycle [14,52], although we found no suitable system to evaluate it in the viral context. Nevertheless, because of the biological importance of this interaction, we believe that it would be worthwhile to solve the structure of NS1 interacting with the viral motif AGCAAAAG. Indeed, in spite of its low affinity, the interaction of the RBD that we observed with aptamer-derived DM01-short (Figure S3) is a promising hint as to the possibility of solving the structure of such a complex and hopefully unveiling a new mode of interaction of NS1 with RNAs.

## Figures and Tables

**Table 1:** Binding of RBD of NS1 from H7N1 to native and truncated RNA aptamers.

Name	nt Sequence / 2D Structure	Length (nt)	RBD Binding	Reference
DM01	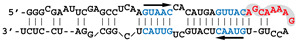	81	++++	[13]
DM01-midi	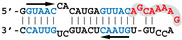	50	++++	this work
DM01-short	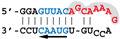	30	+ / - - -	this work
DM01-mot A	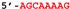	8	-	this work
DM03	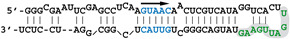	81	++++	[13]
DM03-midi	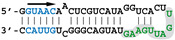	50	++++	this work
DM03-short	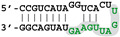	32	+ / - - -	this work
DM03-mot B	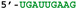	9	-	this work

**Table 2:** Dissociation constant of various RBDs for AWFC01 K_D_ values were extracted from three independent EMSA titration experiments (Figure S5). NA is for “non-applicable”.

RBD.	K_D_ (nM)
H7N1	0.83 ± 0.03
H7N1 R38A-K41A	NA (>50 µM)
H5N1	0.98 ± 0.04
H5N1 R38A-K41A	NA (>100)
H7N9	3.1 ± 0.1
H3N2	3.7 ± 0.1
H17N10	1.5 ± 0.1
pdmH1N1	2.05 ± 0.02

**Table 3:** EC50app for the dissociation of [AWFC01/RBD] by cold RNA competitors. EC50app were determined from dose-response curves as described in Materials and Methods. A representative experiment is shown in Figure S6.

RNA Competitor		EC50app (nM)	
2D-structure	Name	H5N1	H7N1
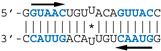	AWFC01	1.0 ± 0.1	1.7 ± 0.1
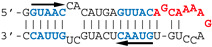	DM01-midi	2.2 ± 0.2	2.7 ± 0.1
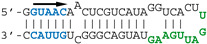	DM03-midi	1.8 ± 0.1	2.8 ± 0.1
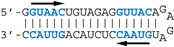	shDM02-GAGA	0.41 ± 0.02	0.24 ± 0.01
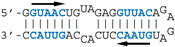	shDM03-GAGA	1.2 ± 0.1	0.64 ± 0.06
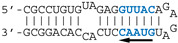	shDM05-GAGA	100 ± 11	ND
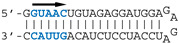	shDM06-GAGA	1.1 ± 0.1	0.85 ± 0.05
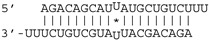	ZKO-RNA	90 ± 4	96 ± 3

The double-stranded GUAAC motif (blue) is emphasized and oriented by the horizontal arrow. The virus specific motifs A and B are shown in red and green, respectively. * depicts the central U*U Hoogsteen base pair in AWFC01 and ZKO-RNA.

**Table 4:** Hydrogen bonds between protein and RNA.

	wt-RBD / AWFC01				aa-RBD / AWFC01				wt-RBD / ZKO *				2ZKO			
	RBD chain A		RBD chain B		RBD chain A		RBD chain B		RBD chain A		RBD chain B		RBD chain A		RBD chain B	
**Atom 1**	**dist.**	Atom 2	**dist.**	Atom 2	**dist.**	Atom 2	**dist.**	Atom 2	**dist.**	Atom 2	**dist.**	Atom 2	**dist.**	Atom 2	**dist.**	Atom 2
**Amino-acid / phosphate H-bonds**																
**H(-1) N**									**3.37**	U19(C)OP1						
**R38 N(ε)**	**2.92 (b)**	C6(D)OP1	**2.76**	C6(C)OP1					**3.18**	G6(D)OP1	**2.98**	G6(C)OP1	**3.47**	G6(D)OP1	**3.42**	G6(C)OP1
**R38 N(η1)**	**3.23 (b)**	C6(D)OP1														
**R38 N(η1)**	**2.99 (b)**	U7(D)OP2	**2.88**	U7(D)OP1					**2.59**	C7(C)OP1	**2.77**	C7(D)OP1	**2.76**	C7(C)OP1	**2.76**	C7(D)OP1
**R38 N(η2)**			**3.26**	C6(C)OP1					**3.18**	G6(D)OP1	**3.16**	G6(C)OP1	**3.43**	G6(D)OP1	**3.35**	G6(C)OP1
**R38 N(η2)**	**2.32 (a)** **2.27 (b)**	U7(C)OP1	**3.06**	U7(C)OP2					**2.96**	C7(D)OP2	**2.77**	C7(C)OP2	**2.83**	C7(D)OP2	**2.87**	C7(C)OP2
**S42 O(γ)**													**2.87**	A5(D)O3’	**2.91**	A5(C)O3’
**Amino-acid / ribose H-bonds**																
**S1 O(γ)**									**2.49**	U17(C)O2’						
**P31 O**	**3.71**	C6(C)O2’	**3.61**	C6(D)O2’	**3.59**	C6(C)O2’	**3.54**	C6(D)O2’	**3.63**	G6(C)O2’	**3.69**	G6(D)O2’			**3.67**	G6(D)O2’
**R35 N(η1)**	**3.54**	C6(C)O2’	**3.66**	C6(D)O2’	**3.55**	C6(C)O2’	**3.51**	C6(D)O2’	**3.44**	G6(C)O2’	**3.38**	G6(D)O2’	**3.14**	G6(C)O2’	**3.22**	G6(D)O2’
**R37 N(η)**			**3.43 (a)**	U7(D)O2’												
**T49 O(γ)**	**3.19**	A4(D)O2’	**3.17**	A4(C)O2’	**3.00**	A4(D)O2’	**3.11**	A4(C)O2’	**2.81**	C4(D)O2’	**3.10**	C4(C)O2’	**2.82**	C4(D)O2’	**2.80**	C4(C)O2’
**T49 O(γ)**	**3.01**	A4(D)O4’	**3.06**	A4(C)O4’	**3.08**	A4(D)O4’	**2.96**	A4(C)O4’	**2.99**	C4(D)O4’	**2.94**	C4(C)O4’	**3.03**	C4(D)O4’	**3.03**	C4(C)O4’

*: a and b (second and fourth columns) refer to the two alternative conformations that were observed for the R38 and R37 side chains. The shaded cells highlight the distinctive asymmetry in the structure of the complex wt-RBD/AWFC01. C or D after the nucleotide number refer to nucleotide chains C and D.

## Supplementary Materials

The following are available online at https://www.mdpi.com/1999-4915/12/9/947/s1, Figure S1: RNA probes used in this study, Figure S2: plaque phenotype of the mutant viruses; Figure S3: Comparative titration of DM01-short, DM01-MotA, DM03-short and DM03-MotB by RBD; Figure S4: Comparative binding properties of selected RBDs with DM01-midi and DM03-midi; Figure S5: Titration curves obtained for the recognition of AWFC01 by various RBDs; Figure S6: EC50app for the displacement of the preformed RBD/AWFC01 by other RNA probes; Figure S7: Comparison of H7N1 NS1 RBD with allele A RBDs; Figure S8: RNA binding interface of NS1 RBD; Figure S9: Conserved positively charged residues of the RNA binding interface; Figure S10: Comparison of the protein backbones in the structure of the three RBD-RNA complexes; Figures S11 and S12: RNA structural parameters from Curves+; Table S1: X-ray data collection and refinement statistics.
