# Supplementary material for: Structure and Sequence Determinants Governing the Interactions of RNAs with Influenza A Virus Non-Structural Protein NS1
Source: Viruses. 2020 Aug 27;12(9):947. doi: 10.3390/v12090947 (PMC7552008; doi:10.3390/v12090947)
Supplement: Supplementary file 1 [file viruses-12-00947-s001.zip › 6SX0_val-report-full_P1.pdf]

Full wwPDB X-ray Structure Validation Report (i)

Sep 25, 2019 – 09:54 am BST

PDB ID	:	6SX0
Title	:	Specific dsRNA recognition by wild type H7N1 NS1 RNA-binding domain
Deposited on	:	2019-09-24
Resolution	:	1.75 Å(reported)

This is a Full wwPDB X-ray Structure Validation Report.

This report is produced by the wwPDB biocuration pipeline after annotation of the structure.

We welcome your comments at *validation@mail.wwpdb.org*
A user guide is available at
https://www.wwpdb.org/validation/2017/XrayValidationReportHelp
with specific help available everywhere you see the (i) symbol.

CONFIDENTIAL VALIDATION REPORT

The following versions of software and data (see references (i)) were used in the production of this report:

MolProbity	:	4.02b-467
Mogul	:	1.8.0 (224370), CSD as540be (2019)
Xtriage (Phenix)	:	1.13
EDS	:	2.4
buster-report	:	1.1.7 (2018)
Percentile statistics	:	20171227.v01 (using entries in the PDB archive December 27th 2017)
Refmac	:	5.8.0158
CCP4	:	7.0 (Gargrove)
Ideal geometry (proteins)	:	Engh & Huber (2001)
Ideal geometry (DNA, RNA)	:	Parkinson et al. (1996)
Validation Pipeline (wwPDB-VP)	:	2.4

1 Overall quality at a glance (i)

The following experimental techniques were used to determine the structure:
X-RAY DIFFRACTION

The reported resolution of this entry is 1.75 Å.

Percentile scores (ranging between 0-100) for global validation metrics of the entry are shown in the following graphic. The table shows the number of entries on which the scores are based.

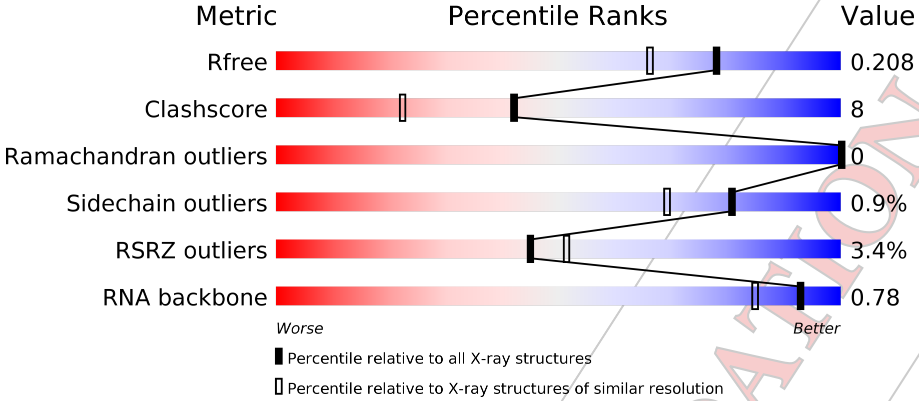

Metric	Whole archive (#Entries)	Similar resolution (#Entries, resolution range(Å))
R_{free}	111664	1952 (1.76-1.76)
Clashscore	122126	2072 (1.76-1.76)
Ramachandran outliers	120053	2050 (1.76-1.76)
Sidechain outliers	120020	2050 (1.76-1.76)
RSRZ outliers	108989	1913 (1.76-1.76)
RNA backbone	2636	1011 (2.50-1.02)

The table below summarises the geometric issues observed across the polymeric chains and their fit to the electron density. The red, orange, yellow and green segments on the lower bar indicate the fraction of residues that contain outliers for >=3, 2, 1 and 0 types of geometric quality criteria. A grey segment represents the fraction of residues that are not modelled. The numeric value for each fraction is indicated below the corresponding segment, with a dot representing fractions <=5% The upper red bar (where present) indicates the fraction of residues that have poor fit to the electron density. The numeric value is given above the bar.

Mol	Chain	Length	Quality of chain
1	A	77	3% / 78% 13% 9%
1	B	77	4% / 82% 10% 8%
2	C	19	5% / 84% 16%
2	D	19	74% 26%

CONFIDENTIAL VALIDATION REPORT

The following table lists non-polymeric compounds, carbohydrate monomers and non-standard residues in protein, DNA, RNA chains that are outliers for geometric or electron-density-fit criteria:

Mol	Type	Chain	Res	Chirality	Geometry	Clashes	Electron density
3	PE4	B	101	-	-	X	-

CONFIDENTIAL VALIDATION REPORT

2 Entry composition (i)

There are 5 unique types of molecules in this entry. The entry contains 2134 atoms, of which 0 are hydrogens and 0 are deuteriums.

In the tables below, the ZeroOcc column contains the number of atoms modelled with zero occupancy, the AltConf column contains the number of residues with at least one atom in alternate conformation and the Trace column contains the number of residues modelled with at most 2 atoms.

- Molecule 1 is a protein called Non-structural protein 1.

Mol	Chain	Residues	Atoms					ZeroOcc	AltConf	Trace
1	A	70	Total<br>558	C<br>350	N<br>100	O<br>102	S<br>6	0	3	0
1	B	71	Total<br>572	C<br>358	N<br>103	O<br>106	S<br>5	0	2	0

There are 10 discrepancies between the modelled and reference sequences:

Chain	Residue	Modelled	Actual	Comment	Reference
A	-3	GLY	-	expression tag	UNP Q1PST0
A	-2	SER	-	expression tag	UNP Q1PST0
A	-1	HIS	-	expression tag	UNP Q1PST0
A	0	MET	-	expression tag	UNP Q1PST0
A	1	SER	-	expression tag	UNP Q1PST0
B	-3	GLY	-	expression tag	UNP Q1PST0
B	-2	SER	-	expression tag	UNP Q1PST0
B	-1	HIS	-	expression tag	UNP Q1PST0
B	0	MET	-	expression tag	UNP Q1PST0
B	1	SER	-	expression tag	UNP Q1PST0

- Molecule 2 is a RNA chain called RNA (5'-R(*GP*GP*UP*AP*AP*CP*UP*GP*UP*UP*AP*CP*AP*GP*UP*UP*AP*CP*C)-3').

Mol	Chain	Residues	Atoms					ZeroOcc	AltConf	Trace
2	C	19	Total<br>388	C<br>175	N<br>64	O<br>131	P<br>18	0	0	0
2	D	19	Total<br>391	C<br>176	N<br>66	O<br>131	P<br>18	0	0	0

- Molecule 3 is 2-{2-[2-(2-{2-[2-(2-ETHOXY-ETHOXY)-ETHOXY]-ETHOXY}-ETHOXY)-ETHOXY]-ETHOXY}-ETHANOL (three-letter code: PE4) (formula: $C_{16}H_{34}O_8$).

CONFIDENTIAL VALIDATION REPORT

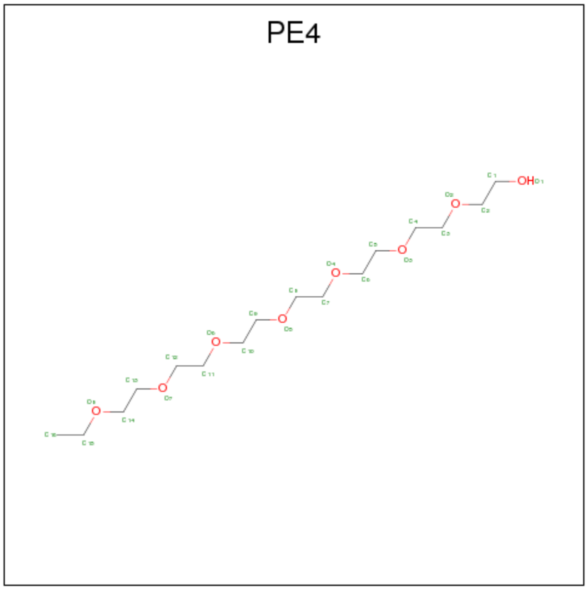

Mol	Chain	Residues	Atoms	ZeroOcc	AltConf
3	A	1	Total C O<br>7 4 3	0	0
3	A	1	Total C O<br>7 4 3	0	0
3	A	1	Total C O<br>11 7 4	0	0
3	B	1	Total C O<br>19 12 7	0	0
3	B	1	Total C O<br>8 5 3	0	0

- Molecule 4 is 2-[N-CYCLOHEXYLAMINO]ETHANE SULFONIC ACID (three-letter code: NHE) (formula: $C_8H_{17}NO_3S$).

CONFIDENTIAL VALIDATION REPORT

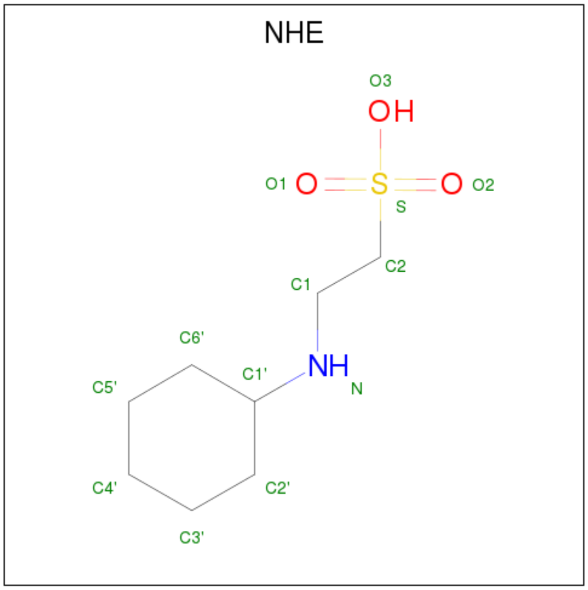

Mol	Chain	Residues	Atoms	ZeroOcc	AltConf
4	A	1	Total C N O S<br>13 8 1 3 1	0	0

- Molecule 5 is water.

Mol	Chain	Residues	Atoms	ZeroOcc	AltConf
5	A	27	Total O<br>27 27	0	0
5	B	33	Total O<br>33 33	0	0
5	C	50	Total O<br>50 50	0	0
5	D	50	Total O<br>50 50	0	0

CONFIDENTIAL VALIDATION REPORT

3 Residue-property plots (i)

These plots are drawn for all protein, RNA and DNA chains in the entry. The first graphic for a chain summarises the proportions of the various outlier classes displayed in the second graphic. The second graphic shows the sequence view annotated by issues in geometryand electron density. Residues are color-coded according to the number of geometric quality criteria for which they contain at least one outlier: green = 0, yellow = 1, orange = 2 and red = 3 or more. A red dot above a residue indicates a poor fit to the electron density (RSRZ > 2). Stretches of 2 or more consecutive residues without any outlier are shown as a green connector. Residues present in the sample, but not in the model, are shown in grey.

- Molecule 1: Non-structural protein 1

Chain A:
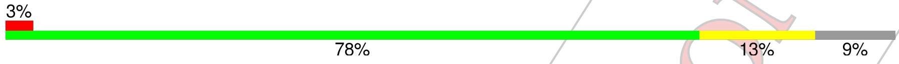

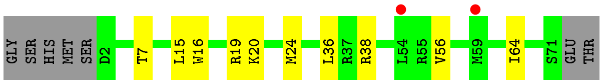

- Molecule 1: Non-structural protein 1

Chain B:
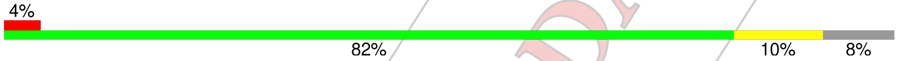

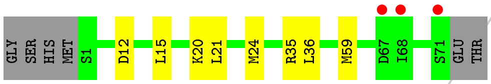

- Molecule 2: RNA (5'-R(*GP*GP*UP*AP*AP*CP*UP*GP*UP*UP*AP*CP*AP*GP*UP*UP *AP*CP*C)-3')

Chain C:
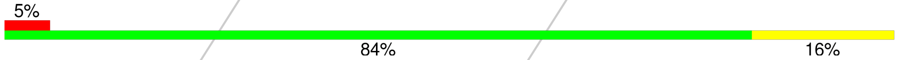

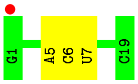

- Molecule 2: RNA (5'-R(*GP*GP*UP*AP*AP*CP*UP*GP*UP*UP*AP*CP*AP*GP*UP*UP *AP*CP*C)-3')

Chain D:
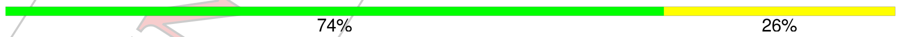

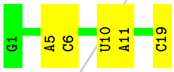

CONFIDENTIAL VALIDATION REPORT

4 Data and refinement statistics (i)

Property	Value	Source
Space group	P 21 21 21	Depositor
Cell constants<br>a, b, c, α, β, γ	49.78Å 60.40Å 87.40Å<br>90.00° 90.00° 90.00°	Depositor
Resolution (Å)	43.26 – 1.75<br>43.26 – 1.75	Depositor<br>EDS
% Data completeness<br>(in resolution range)	99.8 (43.26-1.75)<br>99.9 (43.26-1.75)	Depositor<br>EDS
R_{merge}	0.05	Depositor
R_{sym}	(Not available)	Depositor
$< I/\sigma(I) >$ [1]	1.43 (at 1.75Å)	Xtriage
Refinement program	PHENIX 1.16_3549	Depositor
R, R_{free}	0.171 , 0.203<br>0.177 , 0.208	Depositor<br>DCC
R_{free} test set	1369 reflections (5.03%)	wwPDB-VP
Wilson B-factor (Å^2)	34.7	Xtriage
Anisotropy	0.166	Xtriage
Bulk solvent k_{sol}(e/Å^3), B_{sol}(Å^2)	0.36 , 58.7	EDS
L-test for twinning[2]	$< \|L\| > = 0.50$, $< L^2 > = 0.33$	Xtriage
Estimated twinning fraction	No twinning to report.	Xtriage
F_o,F_c correlation	0.97	EDS
Total number of atoms	2134	wwPDB-VP
Average B, all atoms (Å^2)	42.0	wwPDB-VP

Xtriage's analysis on translational NCS is as follows: *The largest off-origin peak in the Patterson function is 6.42% of the height of the origin peak. No significant pseudotranslation is detected.*

[1] Intensities estimated from amplitudes.

[2] Theoretical values of $< |L| >$, $< L^2 >$ for acentric reflections are 0.5, 0.333 respectively for untwinned datasets, and 0.375, 0.2 for perfectly twinned datasets.

CONFIDENTIAL VALIDATION REPORT

5 Model quality (i)

5.1 Standard geometry (i)

Bond lengths and bond angles in the following residue types are not validated in this section: NHE, PE4

The Z score for a bond length (or angle) is the number of standard deviations the observed value is removed from the expected value. A bond length (or angle) with $|Z| > 5$ is considered an outlier worth inspection. RMSZ is the root-mean-square of all Z scores of the bond lengths (or angles).

Mol	Chain	Bond lengths		Bond angles	
		RMSZ	#$|Z|$ >5	RMSZ	#$|Z|$ >5
1	A	0.36	0/573	0.53	0/768
1	B	0.33	0/584	0.51	0/781
2	C	0.45	0/432	0.81	0/670
2	D	0.45	0/436	0.85	0/677
All	All	0.39	0/2025	0.68	0/2896

There are no bond length outliers.

There are no bond angle outliers.

There are no chirality outliers.

There are no planarity outliers.

5.2 Too-close contacts (i)

In the following table, the Non-H and H(model) columns list the number of non-hydrogen atoms and hydrogen atoms in the chain respectively. The H(added) column lists the number of hydrogen atoms added and optimized by MolProbity. The Clashes column lists the number of clashes within the asymmetric unit, whereas Symm-Clashes lists symmetry related clashes.

Mol	Chain	Non-H	H(model)	H(added)	Clashes	Symm-Clashes
1	A	558	0	563	12	0
1	B	572	0	584	8	0
2	C	388	0	200	3	0
2	D	391	0	200	3	0
3	A	25	0	31	1	0
3	B	27	0	33	11	0
4	A	13	0	17	2	0
5	A	27	0	0	2	0
5	B	33	0	0	0	0

Continued on next page...

CONFIDENTIAL VALIDATION REPORT

Continued from previous page...

Mol	Chain	Non-H	H(model)	H(added)	Clashes	Symm-Clashes
5	C	50	0	0	0	0
5	D	50	0	0	0	0
All	All	2134	0	1628	27	0

The all-atom clashscore is defined as the number of clashes found per 1000 atoms (including hydrogen atoms). The all-atom clashscore for this structure is 8.

All (27) close contacts within the same asymmetric unit are listed below, sorted by their clash magnitude.

Atom-1	Atom-2	Interatomic distance (Å)	Clash overlap (Å)
1:B:12:ASP:HA	3:B:101:PE4:H121	1.56	0.87
1:A:19:ARG:HH11	3:B:101:PE4:H42	1.51	0.76
1:B:15:LEU:HB2	3:B:101:PE4:H122	1.68	0.75
4:A:104:NHE:O1	5:A:201:HOH:O	2.06	0.73
1:A:38[B]:ARG:NH2	2:C:7:U:OP1	2.27	0.67
1:B:21:LEU:HD23	1:B:24:MET:HE3	1.85	0.59
3:B:101:PE4:H61	3:B:101:PE4:O7	2.03	0.58
1:A:19:ARG:NH1	3:B:101:PE4:H62	2.20	0.56
1:A:56:VAL:HG11	4:A:104:NHE:HC11	1.88	0.55
1:B:15:LEU:HB2	3:B:101:PE4:C12	2.36	0.55
1:A:38[A]:ARG:NH2	2:C:7:U:OP1	2.32	0.53
1:A:19:ARG:NH1	3:B:101:PE4:H42	2.22	0.50
1:A:20:LYS:O	1:A:24:MET:HG3	2.11	0.50
3:B:101:PE4:H102	3:B:101:PE4:H82	1.54	0.48
1:A:7:THR:HG23	1:A:64:ILE:HD13	1.97	0.47
1:B:15:LEU:CB	3:B:101:PE4:H122	2.43	0.47
1:A:38[B]:ARG:NH2	5:A:203:HOH:O	2.46	0.46
1:A:15:LEU:HB2	3:B:101:PE4:H41	1.98	0.45
1:B:20:LYS:HA	1:B:36:LEU:HD21	1.99	0.45
2:C:5:A:H2'	2:C:6:C:O4'	2.16	0.44
2:D:10:U:H2'	2:D:11:A:C8	2.53	0.44
1:A:16:TRP:CE3	3:A:102:PE4:H32	2.53	0.44
2:D:5:A:H2'	2:D:6:C:O4'	2.16	0.44
1:B:21:LEU:HD23	1:B:24:MET:CE	2.48	0.43
3:B:102:PE4:H51	3:B:102:PE4:H31	1.71	0.42
1:A:20:LYS:HA	1:A:36:LEU:HD21	2.01	0.41
1:B:35:ARG:HD3	2:D:6:C:H4'	2.02	0.41

There are no symmetry-related clashes.

CONFIDENTIAL VALIDATION REPORT

5.3 Torsion angles (i)

5.3.1 Protein backbone (i)

In the following table, the Percentiles column shows the percent Ramachandran outliers of the chain as a percentile score with respect to all X-ray entries followed by that with respect to entries of similar resolution.

The Analysed column shows the number of residues for which the backbone conformation was analysed, and the total number of residues.

Mol	Chain	Analysed	Favoured	Allowed	Outliers	Percentiles	
1	A	71/77 (92%)	71 (100%)	0	0	100	100
1	B	71/77 (92%)	71 (100%)	0	0	100	100
All	All	142/154 (92%)	142 (100%)	0	0	100	100

There are no Ramachandran outliers to report.

5.3.2 Protein sidechains (i)

In the following table, the Percentiles column shows the percent sidechain outliers of the chain as a percentile score with respect to all X-ray entries followed by that with respect to entries of similar resolution.

The Analysed column shows the number of residues for which the sidechain conformation was analysed, and the total number of residues.

Mol	Chain	Analysed	Rotameric	Outliers	Percentiles	
1	A	60/69 (87%)	60 (100%)	0	100	100
1	B	62/69 (90%)	61 (98%)	1 (2%)	65	48
All	All	122/138 (88%)	121 (99%)	1 (1%)	81	74

All (1) residues with a non-rotameric sidechain are listed below:

Mol	Chain	Res	Type
1	B	59	MET

Some sidechains can be flipped to improve hydrogen bonding and reduce clashes. There are no such sidechains identified.

5.3.3 RNA (i)

CONFIDENTIAL VALIDATION REPORT

Mol	Chain	Analysed	Backbone Outliers	Pucker Outliers
2	C	18/19 (94%)	0	0
2	D	18/19 (94%)	1 (5%)	0
All	All	36/38 (94%)	1 (2%)	0

All (1) RNA backbone outliers are listed below:

Mol	Chain	Res	Type
2	D	19	C

There are no RNA pucker outliers to report.

5.4 Non-standard residues in protein, DNA, RNA chains (i)

There are no non-standard protein/DNA/RNA residues in this entry.

5.5 Carbohydrates (i)

There are no carbohydrates in this entry.

5.6 Ligand geometry (i)

6 ligands are modelled in this entry.

In the following table, the Counts columns list the number of bonds (or angles) for which Mogul statistics could be retrieved, the number of bonds (or angles) that are observed in the model and the number of bonds (or angles) that are defined in the Chemical Component Dictionary. The Link column lists molecule types, if any, to which the group is linked. The Z score for a bond length (or angle) is the number of standard deviations the observed value is removed from the expected value. A bond length (or angle) with $|Z| > 2$ is considered an outlier worth inspection. RMSZ is the root-mean-square of all Z scores of the bond lengths (or angles).

Mol	Type	Chain	Res	Link	Bond lengths			Bond angles		
					Counts	RMSZ	$\#\|Z\| > 2$	Counts	RMSZ	$\#\|Z\| > 2$
3	PE4	A	101	-	6,6,23	0.49	0	5,5,22	0.25	0
3	PE4	A	102	-	6,6,23	0.49	0	5,5,22	0.32	0
3	PE4	A	103	-	10,10,23	0.51	0	9,9,22	0.24	0
4	NHE	A	104	-	13,13,13	1.45	3 (23%)	16,17,17	1.99	4 (25%)
3	PE4	B	101	-	18,18,23	0.57	0	17,17,22	0.43	0
3	PE4	B	102	-	7,7,23	0.48	0	6,6,22	0.29	0

In the following table, the Chirals column lists the number of chiral outliers, the number of chiral

CONFIDENTIAL VALIDATION REPORT

centers analysed, the number of these observed in the model and the number defined in the Chemical Component Dictionary. Similar counts are reported in the Torsion and Rings columns. '-' means no outliers of that kind were identified.

Mol	Type	Chain	Res	Link	Chirals	Torsions	Rings
3	PE4	A	101	-	-	1/4/4/21	-
3	PE4	A	102	-	-	2/4/4/21	-
3	PE4	A	103	-	-	5/8/8/21	-
4	NHE	A	104	-	-	4/7/15/15	0/1/1/1
3	PE4	B	101	-	-	10/16/16/21	-
3	PE4	B	102	-	-	2/5/5/21	-

All (3) bond length outliers are listed below:

Mol	Chain	Res	Type	Atoms	Z	Observed(Å)	Ideal(Å)
4	A	104	NHE	C2-S	3.75	1.82	1.77
4	A	104	NHE	O2-S	2.21	1.51	1.45
4	A	104	NHE	O1-S	2.16	1.51	1.45

All (4) bond angle outliers are listed below:

Mol	Chain	Res	Type	Atoms	Z	Observed(^{o})	Ideal(^{o})
4	A	104	NHE	O2-S-C2	4.22	111.99	106.92
4	A	104	NHE	O1-S-C2	3.81	111.50	106.92
4	A	104	NHE	O3-S-O2	-3.47	102.80	111.27
4	A	104	NHE	O3-S-C2	2.65	110.05	105.77

There are no chirality outliers.

All (24) torsion outliers are listed below:

Mol	Chain	Res	Type	Atoms
4	A	104	NHE	N-C1-C2-S
3	B	102	PE4	C3-C4-O3-C5
3	B	102	PE4	O2-C3-C4-O3
3	B	101	PE4	O4-C7-C8-O5
3	B	101	PE4	C10-C9-O5-C8
3	A	103	PE4	O1-C1-C2-O2
4	A	104	NHE	C2-C1-N-C1'
4	A	104	NHE	C6'-C1'-N-C1
3	B	101	PE4	O3-C5-C6-O4
3	B	101	PE4	C9-C10-O6-C11
3	B	101	PE4	C1-C2-O2-C3

Continued on next page...

CONFIDENTIAL VALIDATION REPORT

Continued from previous page...

Mol	Chain	Res	Type	Atoms
3	A	102	PE4	C1-C2-O2-C3
3	B	101	PE4	C4-C3-O2-C2
3	A	103	PE4	C3-C4-O3-C5
3	B	101	PE4	C12-C11-O6-C10
3	A	103	PE4	C4-C3-O2-C2
3	B	101	PE4	C8-C7-O4-C6
3	B	101	PE4	C7-C8-O5-C9
3	A	103	PE4	O3-C5-C6-O4
3	A	102	PE4	C4-C3-O2-C2
4	A	104	NHE	C2'-C1'-N-C1
3	B	101	PE4	C6-C5-O3-C4
3	A	103	PE4	C1-C2-O2-C3
3	A	101	PE4	C4-C3-O2-C2

There are no ring outliers.

4 monomers are involved in 14 short contacts:

Mol	Chain	Res	Type	Clashes	Symm-Clashes
3	A	102	PE4	1	0
4	A	104	NHE	2	0
3	B	101	PE4	10	0
3	B	102	PE4	1	0

The following is a two-dimensional graphical depiction of Mogul quality analysis of bond lengths, bond angles, torsion angles, and ring geometry for all instances of the Ligand of Interest. In addition, ligands with molecular weight > 250 and outliers as shown on the validation Tables will also be included. For torsion angles, if less then 5% of the Mogul distribution of torsion angles is within 10 degrees of the torsion angle in question, then that torsion angle is considered an outlier. Any bond that is central to one or more torsion angles identified as an outlier by Mogul will be highlighted in the graph. For rings, the root-mean-square deviation (RMSD) between the ring in question and similar rings identified by Mogul is calculated over all ring torsion angles. If the average RMSD is greater than 60 degrees and the minimal RMSD between the ring in question and any Mogul-identified rings is also greater than 60 degrees, then that ring is considered an outlier. The outliers are highlighted in purple. The color gray indicates Mogul did not find sufficient equivalents in the CSD to analyse the geometry.

CONFIDENTIAL VALIDATION REPORT

Ligand PE4 A 101

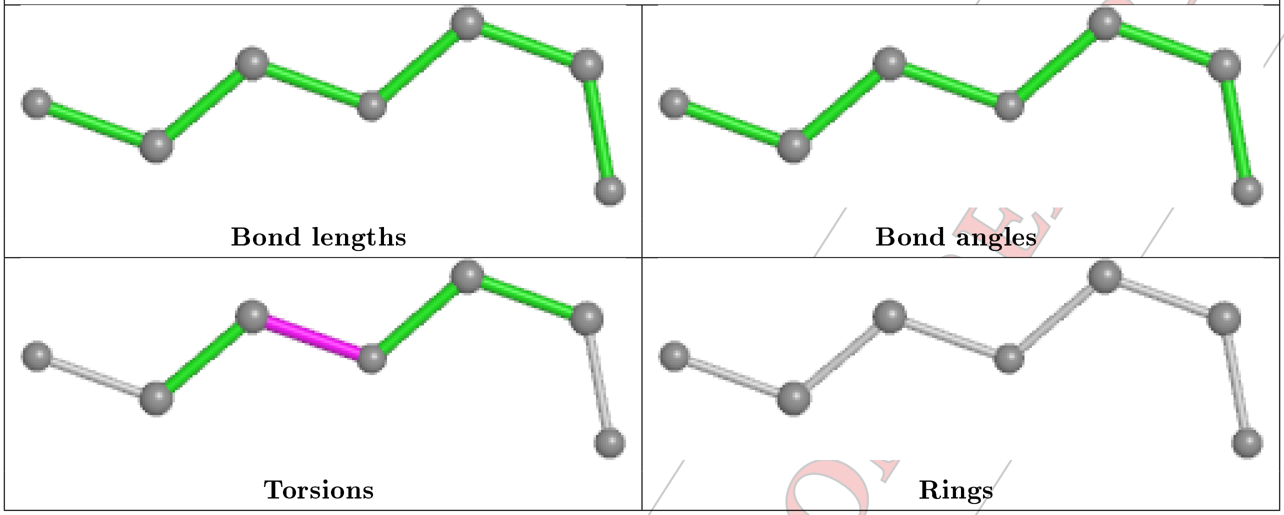

Ligand PE4 A 102

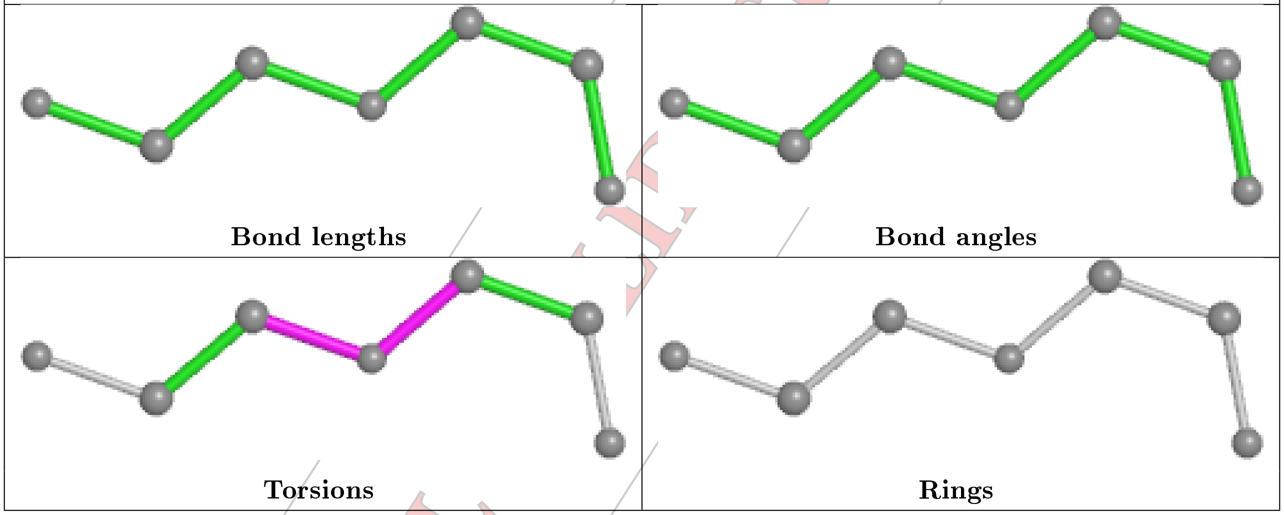

Ligand PE4 A 103

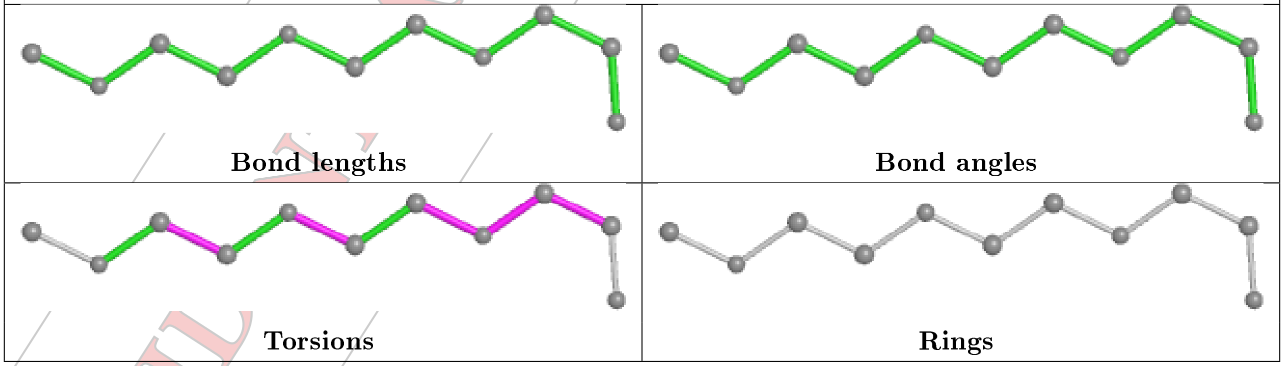

Ligand PE4 B 101

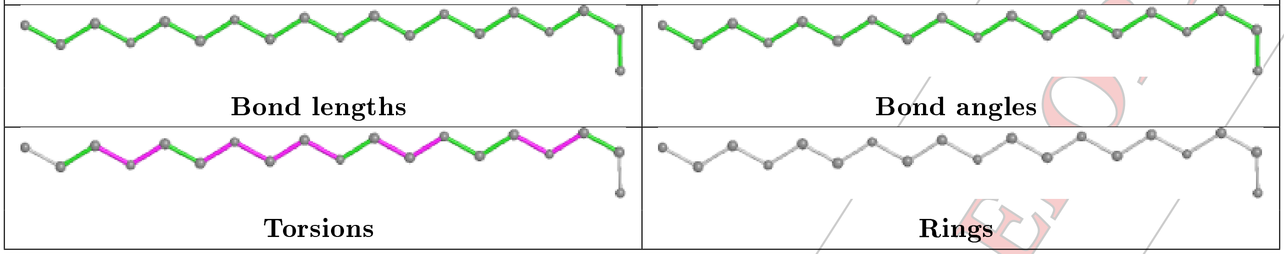

Ligand PE4 B 102

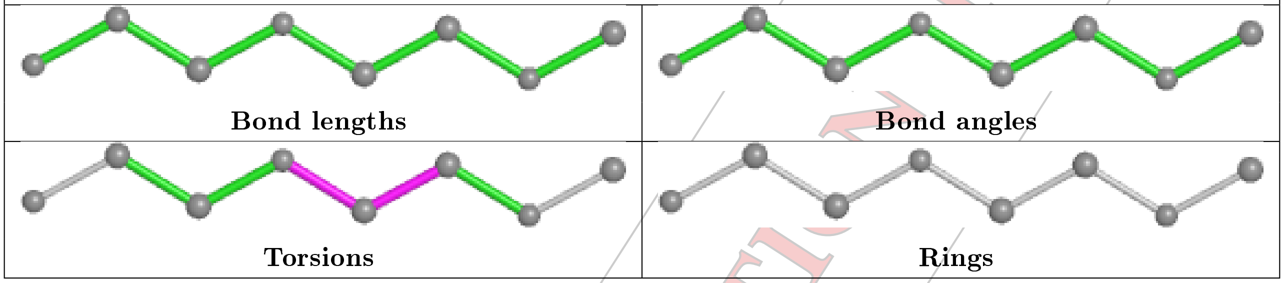

5.7 Other polymers ⓘ

There are no such residues in this entry.

5.8 Polymer linkage issues ⓘ

There are no chain breaks in this entry.

CONFIDENTIAL VALIDATION REPORT

6 Fit of model and data (i)

6.1 Protein, DNA and RNA chains (i)

In the following table, the column labelled '#RSRZ> 2' contains the number (and percentage) of RSRZ outliers, followed by percent RSRZ outliers for the chain as percentile scores relative to all X-ray entries and entries of similar resolution. The OWAB column contains the minimum, median, 95th percentile and maximum values of the occupancy-weighted average B-factor per residue. The column labelled 'Q< 0.9' lists the number of (and percentage) of residues with an average occupancy less than 0.9.

Mol	Chain	Analysed	<RSRZ>	#RSRZ>2			OWAB(Å^2)	Q<0.9
1	A	70/77 (90%)	0.48	2 (2%)	51	57	29, 35, 58, 85	0
1	B	71/77 (92%)	0.51	3 (4%)	36	42	28, 35, 61, 94	0
2	C	19/19 (100%)	0.24	1 (5%)	26	33	31, 37, 76, 96	0
2	D	19/19 (100%)	0.11	0	100	100	30, 37, 69, 83	0
All	All	179/192 (93%)	0.43	6 (3%)	45	51	28, 36, 64, 96	0

All (6) RSRZ outliers are listed below:

Mol	Chain	Res	Type	RSRZ
2	C	1	G	5.0
1	B	68	ILE	2.9
1	A	59[A]	MET	2.8
1	B	67	ASP	2.7
1	A	54	LEU	2.2
1	B	71	SER	2.1

6.2 Non-standard residues in protein, DNA, RNA chains (i)

There are no non-standard protein/DNA/RNA residues in this entry.

6.3 Carbohydrates (i)

There are no carbohydrates in this entry.

6.4 Ligands (i)

In the following table, the Atoms column lists the number of modelled atoms in the group and the number defined in the chemical component dictionary. The B-factors column lists the minimum,

CONFIDENTIAL VALIDATION REPORT

median, 95th percentile and maximum values of B factors of atoms in the group. The column labelled 'Q< 0.9' lists the number of atoms with occupancy less than 0.9.

Mol	Type	Chain	Res	Atoms	RSCC	RSR	B-factors($Å^2$)	Q<0.9
3	PE4	A	103	11/24	0.52	0.28	57,67,78,79	0
3	PE4	B	101	19/24	0.70	0.21	38,52,69,69	0
3	PE4	B	102	8/24	0.76	0.17	61,65,74,79	0
3	PE4	A	102	7/24	0.79	0.21	57,65,80,80	0
4	NHE	A	104	13/13	0.92	0.25	59,71,80,85	0
3	PE4	A	101	7/24	0.93	0.14	48,56,71,78	0

The following is a graphical depiction of the model fit to experimental electron density of all instances of the Ligand of Interest. In addition, ligands with molecular weight > 250 and outliers as shown on the geometry validation Tables will also be included. Each fit is shown from different orientation to approximate a three-dimensional view.

CONFIDENTIAL VALIDATION REPORT

Electron density around PE4 A 103:

$2mF_o$-DF_c (at 0.7 rmsd) in gray
mF_o-DF_c (at 3 rmsd) in purple (negative)
and green (positive)

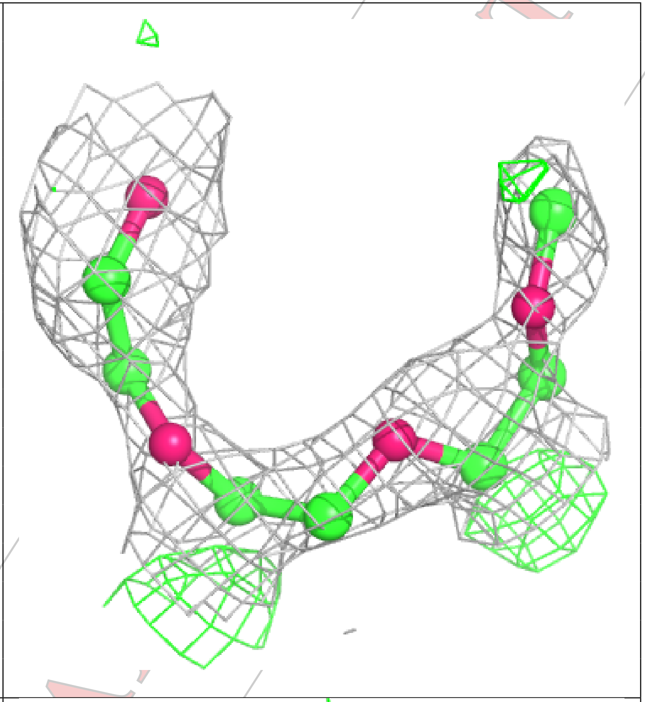

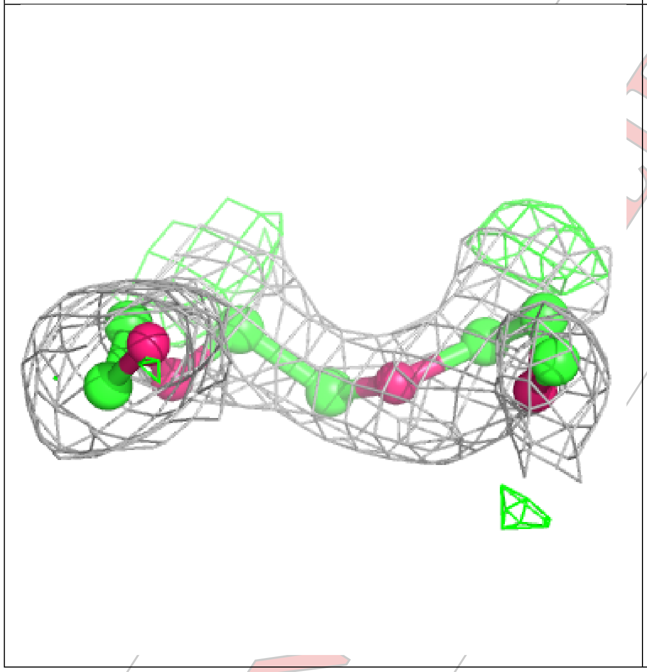

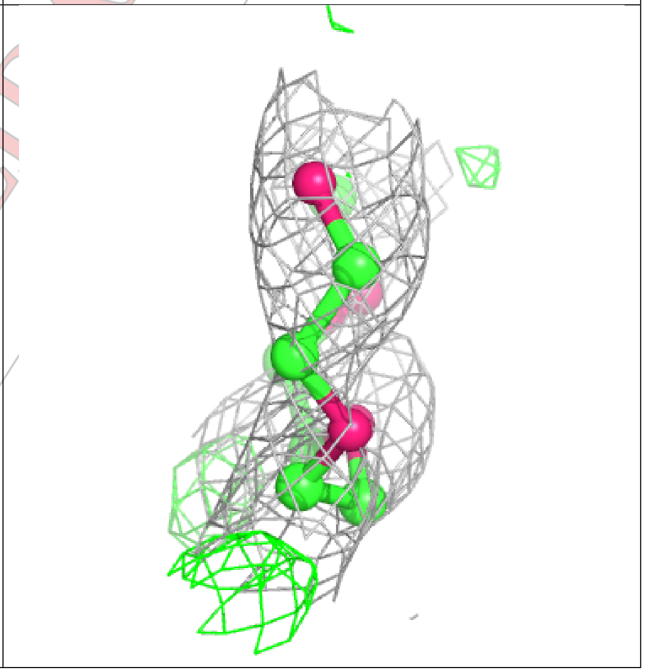

CONFIDENTIAL

Electron density around PE4 B 101:

$2mF_o$-DF_c (at 0.7 rmsd) in gray
mF_o-DF_c (at 3 rmsd) in purple (negative)
and green (positive)

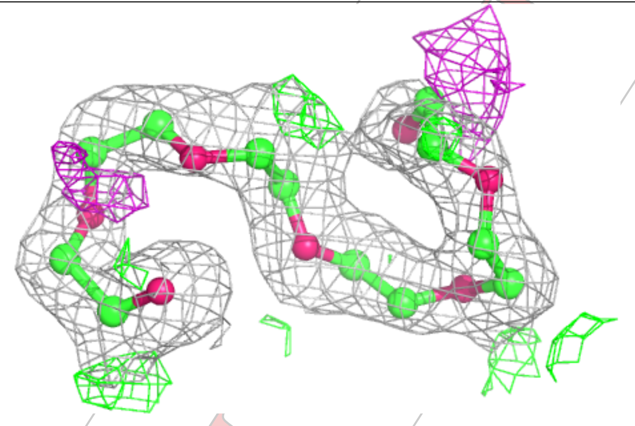

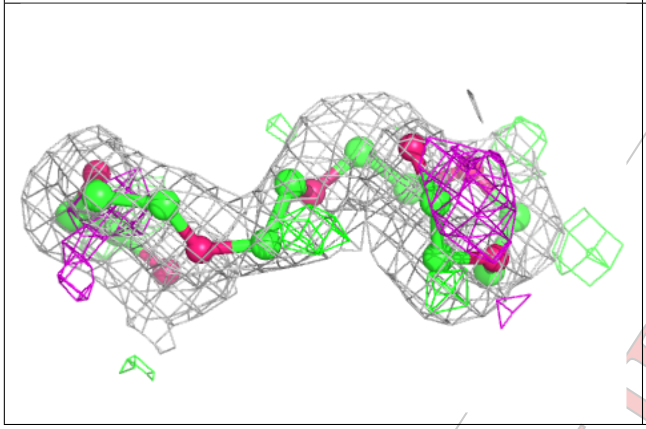

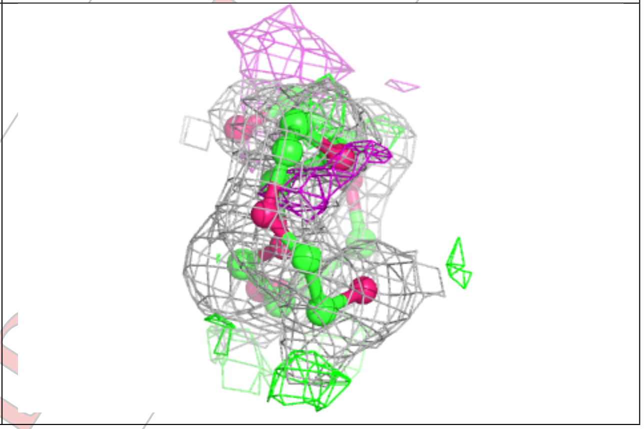

Electron density around PE4 B 102:

$2mF_o$-DF_c (at 0.7 rmsd) in gray
mF_o-DF_c (at 3 rmsd) in purple (negative)
and green (positive)

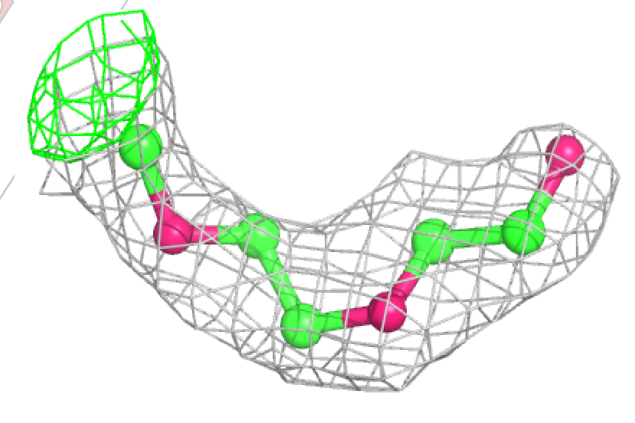

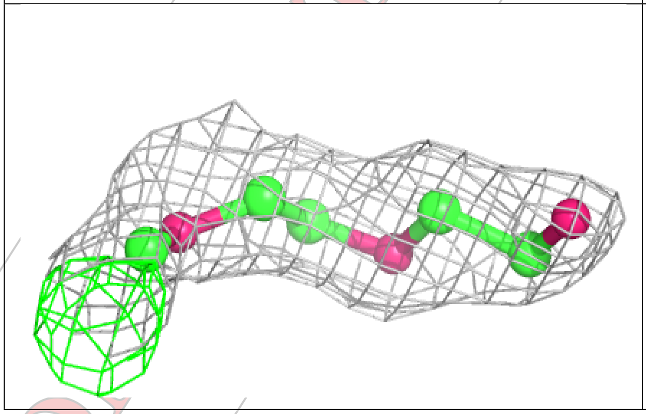

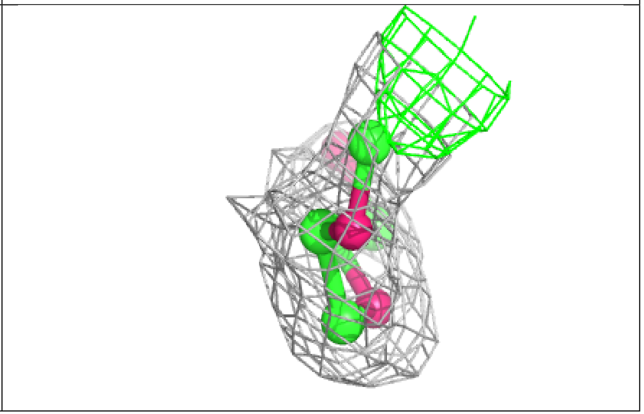

Electron density around PE4 A 102:

2mF$_o$-DF$_c$ (at 0.7 rmsd) in gray
mF$_o$-DF$_c$ (at 3 rmsd) in purple (negative)
and green (positive)

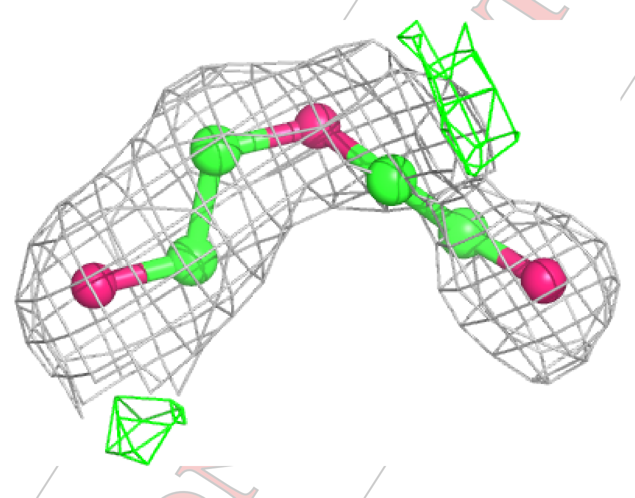

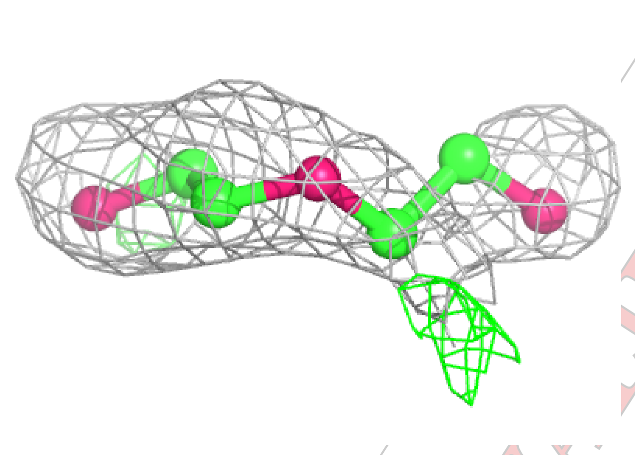

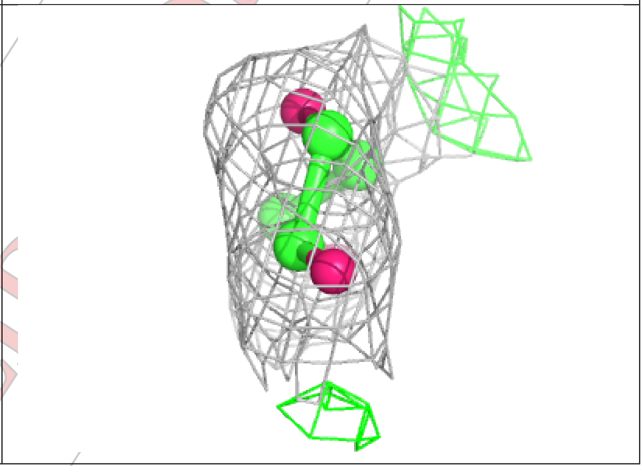

CONFIDENTIAL VALIDATION REPORT

Electron density around PE4 A 101:

$2mF_o$-DF_c (at 0.7 rmsd) in gray
mF_o-DF_c (at 3 rmsd) in purple (negative)
and green (positive)

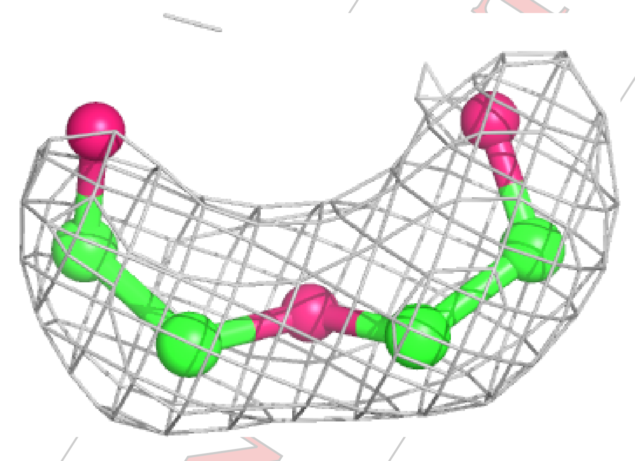

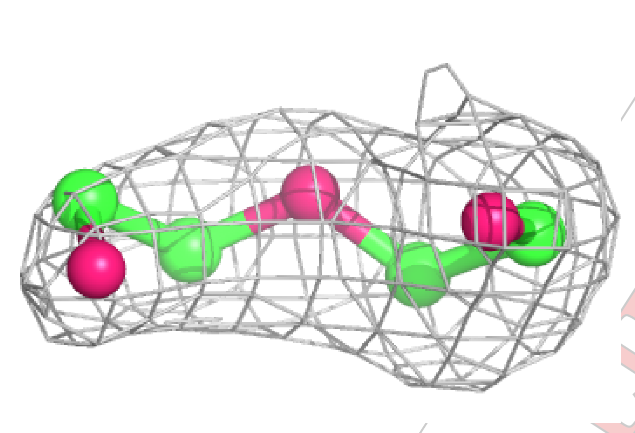

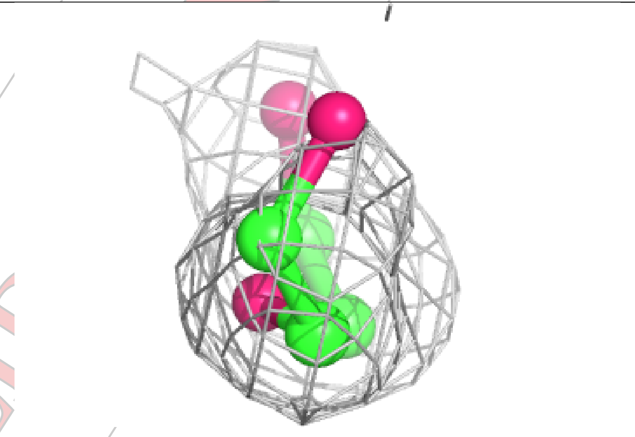

6.5 Other polymers ⓘ

There are no such residues in this entry.
