# Supplementary material for: Structure and Sequence Determinants Governing the Interactions of RNAs with Influenza A Virus Non-Structural Protein NS1
Source: Viruses. 2020 Aug 27;12(9):947. doi: 10.3390/v12090947 (PMC7552008; doi:10.3390/v12090947)
Supplement: Supplementary file 1 [file viruses-12-00947-s001.zip › 6ZLC_val-report-full_P1.pdf]

Preliminary Full wwPDB X-ray Structure Validation Report (i)

Jun 30, 2020 – 10:17 AM BST

Deposition ID : D_1292109703

This is a Preliminary Full wwPDB X-ray Structure Validation Report.

This report is produced by the wwPDB Deposition System during initial deposition but before annotation of the structure.

We welcome your comments at *validation@mail.wwpdb.org*
A user guide is available at
https://www.wwpdb.org/validation/2017/XrayValidationReportHelp
with specific help available everywhere you see the (i) symbol.

PRELIMINARY VALIDATION REPORT

The following versions of software and data (see references (i)) were used in the production of this report:

MolProbity	:	4.02b-467
Mogul	:	1.8.5 (274361), CSD as541be (2020)
Xtriage (Phenix)	:	1.13
EDS	:	2.13.dev1
Percentile statistics	:	20191225.v01 (using entries in the PDB archive December 25th 2019)
Refmac	:	5.8.0158
CCP4	:	7.0.044 (Gargrove)
Ideal geometry (proteins)	:	Engh & Huber (2001)
Ideal geometry (DNA, RNA)	:	Parkinson et al. (1996)
Validation Pipeline (wwPDB-VP)	:	2.13.dev1

1 Overall quality at a glance (i)

The following experimental techniques were used to determine the structure:
X-RAY DIFFRACTION

The reported resolution of this entry is 2.30 Å.

Percentile scores (ranging between 0-100) for global validation metrics of the entry are shown in the following graphic. The table shows the number of entries on which the scores are based.

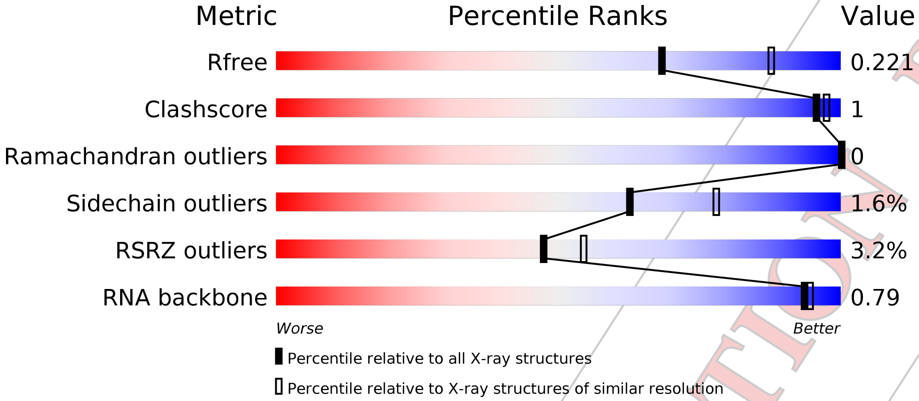

Metric	Whole archive (#Entries)	Similar resolution (#Entries, resolution range(Å))
R_{free}	130704	5042 (2.30-2.30)
Clashscore	141614	5643 (2.30-2.30)
Ramachandran outliers	138981	5575 (2.30-2.30)
Sidechain outliers	138945	5575 (2.30-2.30)
RSRZ outliers	127900	4938 (2.30-2.30)
RNA backbone	3102	1090 (2.70-1.90)

The table below summarises the geometric issues observed across the polymeric chains and their fit to the electron density. The red, orange, yellow and green segments on the lower bar indicate the fraction of residues that contain outliers for >=3, 2, 1 and 0 types of geometric quality criteria respectively. A grey segment represents the fraction of residues that are not modelled. The numeric value for each fraction is indicated below the corresponding segment, with a dot representing fractions <=5% The upper red bar (where present) indicates the fraction of residues that have poor fit to the electron density. The numeric value is given above the bar.

Mol	Chain	Length	Quality of chain
1	A	77	4% / 95% 5%
2	B	72	4% / 93% 7%
3	C	19	95% 5%
3	D	19	100%

PRELIMINARY VALIDATION REPORT

2 Entry composition (i)

There are 6 unique types of molecules in this entry. The entry contains 2045 atoms, of which 0 are hydrogens and 0 are deuteriums.

In the tables below, the ZeroOcc column contains the number of atoms modelled with zero occupancy, the AltConf column contains the number of residues with at least one atom in alternate conformation and the Trace column contains the number of residues modelled with at most 2 atoms.

- Molecule 1 is a protein.

Mol	Chain	Residues	Atoms					ZeroOcc	AltConf	Trace
			Total	C	N	O	S			
1	A	77	595	367	107	115	6	0	0	0

- Molecule 2 is a protein.

Mol	Chain	Residues	Atoms					ZeroOcc	AltConf	Trace
			Total	C	N	O	S			
2	B	72	570	353	103	109	5	0	0	0

- Molecule 3 is a RNA chain called RNA (5'-R(*AP*GP*AP*CP*AP*GP*CP*AP*UP*UP*AP*UP*GP*CP*UP*GP*UP*CP*U)-3').

Mol	Chain	Residues	Atoms					ZeroOcc	AltConf	Trace
			Total	C	N	O	P			
3	C	19	399	180	69	132	18	0	0	0
			Total	C	N	O	P			
3	D	19	399	180	69	132	18	0	0	0

- Molecule 4 is SULFATE ION (three-letter code: SO4) (formula: O_4S).

PRELIMINARY VALIDATION REPORT

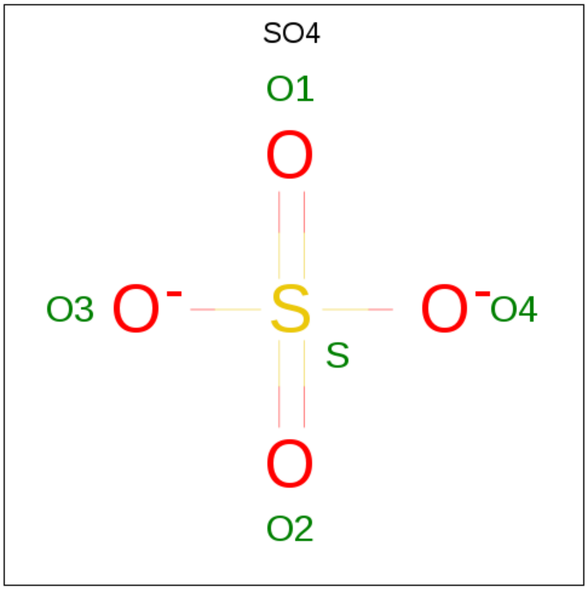

Mol	Chain	Residues	Atoms	ZeroOcc	AltConf
4	E	1	Total O S<br>5 4 1	0	0
4	E	1	Total O S<br>5 4 1	0	0

- Molecule 5 is NITRATE ION (three-letter code: NO3) (formula: NO_3).

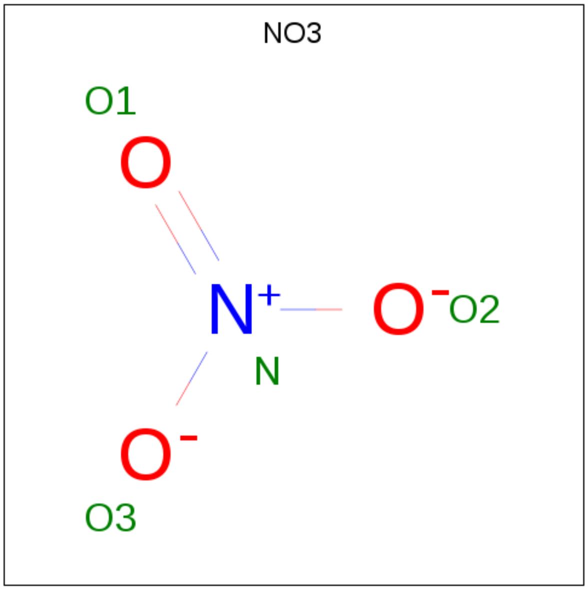

Mol	Chain	Residues	Atoms	ZeroOcc	AltConf
5	F	1	Total N O<br>4 1 3	0	0
5	F	1	Total N O<br>4 1 3	0	0

Continued on next page...

PRELIMINARY VALIDATION REPORT

Continued from previous page...

Mol	Chain	Residues	Atoms	ZeroOcc	AltConf
5	F	1	Total N O<br>4 1 3	0	0
5	F	1	Total N O<br>4 1 3	0	0

- Molecule 6 is water.

Mol	Chain	Residues	Atoms	ZeroOcc	AltConf
6	S	56	Total O<br>56 56	0	0

PRELIMINARY VALIDATION REPORT

3 Residue-property plots (i)

These plots are drawn for all protein, RNA, DNA and oligosaccharide chains in the entry. The first graphic for a chain summarises the proportions of the various outlier classes displayed in the second graphic. The second graphic shows the sequence view annotated by issues in geometry and electron density. Residues are color-coded according to the number of geometric quality criteria for which they contain at least one outlier: green = 0, yellow = 1, orange = 2 and red = 3 or more. A red dot above a residue indicates a poor fit to the electron density (RSRZ > 2). Stretches of 2 or more consecutive residues without any outlier are shown as a green connector. Residues present in the sample, but not in the model, are shown in grey.

- Molecule 1:

Chain A:

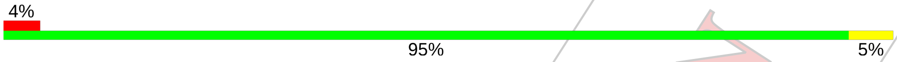

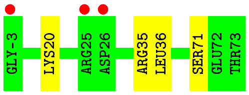

- Molecule 2:

Chain B:

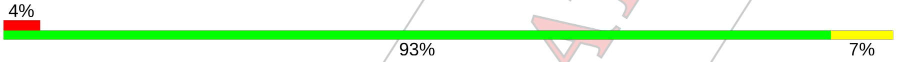

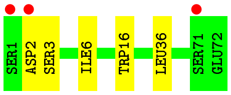

- Molecule 3: RNA (5'-R(*AP*GP*AP*CP*AP*GP*CP*AP*UP*UP*AP*UP*GP*CP*UP*GP *UP*CP*U)-3')

Chain C:

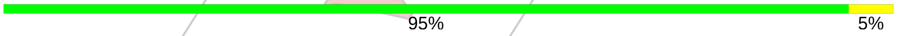

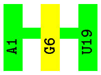

- Molecule 3: RNA (5'-R(*AP*GP*AP*CP*AP*GP*CP*AP*UP*UP*AP*UP*GP*CP*UP*GP *UP*CP*U)-3')

Chain D:

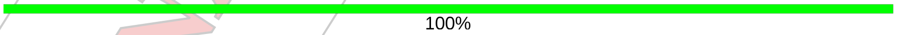

There are no outlier residues recorded for this chain.

PRELIMINARY VALIDATION REPORT

4 Data and refinement statistics (i)

Property	Value	Source
Space group	P 41 21 2	Depositor
Cell constants<br>a, b, c, α, β, γ	103.60Å 103.60Å 102.21Å<br>90.00° 90.00° 90.00°	Depositor
Resolution (Å)	46.33 – 2.30<br>46.33 – 2.30	Depositor<br>EDS
% Data completeness<br>(in resolution range)	100.0 (46.33-2.30)<br>93.6 (46.33-2.30)	Depositor<br>EDS
R_{merge}	(Not available)	Depositor
R_{sym}	(Not available)	Depositor
$< I/\sigma(I) >$ [1]	0.96 (at 2.29Å)	Xtriage
Refinement program	phenix.refine 1.16_3549, PHENIX 1.16_3549	Depositor
R, R_{free}	0.191 , 0.221<br>0.191 , 0.221	Depositor<br>DCC
R_{free} test set	1233 reflections (4.87%)	wwPDB-VP
Wilson B-factor (Å^2)	56.2	Xtriage
Anisotropy	0.038	Xtriage
Bulk solvent k_{sol}(e/Å^3), B_{sol}(Å^2)	0.35 , 51.1	EDS
L-test for twinning[2]	$< \|L\| >$ = 0.50, $< L^2 >$ = 0.33	Xtriage
Estimated twinning fraction	0.009 for -h,-l,-k<br>0.000 for l,-k,h	Xtriage
F_o,F_c correlation	0.96	EDS
Total number of atoms	2045	wwPDB-VP
Average B, all atoms (Å^2)	67.0	wwPDB-VP

Xtriage's analysis on translational NCS is as follows: *The largest off-origin peak in the Patterson function is 5.09% of the height of the origin peak. No significant pseudotranslation is detected.*

[1] Intensities estimated from amplitudes.

[2] Theoretical values of $< |L| >$, $< L^2 >$ for acentric reflections are 0.5, 0.333 respectively for untwinned datasets, and 0.375, 0.2 for perfectly twinned datasets.

PRELIMINARY VALIDATION REPORT

5 Model quality (i)

5.1 Standard geometry (i)

Bond lengths and bond angles in the following residue types are not validated in this section: SO4, NO3

The Z score for a bond length (or angle) is the number of standard deviations the observed value is removed from the expected value. A bond length (or angle) with $|Z| > 5$ is considered an outlier worth inspection. RMSZ is the root-mean-square of all Z scores of the bond lengths (or angles).

Mol	Chain	Bond lengths		Bond angles	
		RMSZ	#|Z| >5	RMSZ	#|Z| >5
1	A	0.31	0/602	0.47	0/806
2	B	0.32	0/576	0.47	0/772
3	C	0.33	0/445	0.76	0/691
3	D	0.35	0/445	0.80	0/691
All	All	0.32	0/2068	0.64	0/2960

There are no bond length outliers.

There are no bond angle outliers.

There are no chirality outliers.

There are no planarity outliers.

5.2 Too-close contacts (i)

In the following table, the Non-H and H(model) columns list the number of non-hydrogen atoms and hydrogen atoms in the chain respectively. The H(added) column lists the number of hydrogen atoms added and optimized by MolProbity. The Clashes column lists the number of clashes within the asymmetric unit, whereas Symm-Clashes lists symmetry related clashes.

Mol	Chain	Non-H	H(model)	H(added)	Clashes	Symm-Clashes
1	A	595	0	583	2	0
2	B	570	0	569	2	0
3	C	399	0	205	1	0
3	D	399	0	205	0	0
4	E	10	0	0	0	0
5	F	16	0	0	0	0
6	S	56	0	0	0	0
All	All	2045	0	1562	4	0

The all-atom clashscore is defined as the number of clashes found per 1000 atoms (including

PRELIMINARY VALIDATION REPORT

hydrogen atoms). The all-atom clashscore for this structure is 1.

All (4) close contacts within the same asymmetric unit are listed below, sorted by their clash magnitude.

Atom-1	Atom-2	Interatomic distance (Å)	Clash overlap (Å)
1:A:35:ARG:HH11	3:C:6:G:H4'	1.76	0.51
2:B:2:ASP:O	2:B:6:ILE:HG12	2.14	0.47
1:A:20:LYS:HA	1:A:36:LEU:HD21	2.00	0.43
2:B:16:TRP:NE1	2:B:36:LEU:HD12	2.34	0.42

There are no symmetry-related clashes.

5.3 Torsion angles (i)

5.3.1 Protein backbone (i)

In the following table, the Percentiles column shows the percent Ramachandran outliers of the chain as a percentile score with respect to all X-ray entries followed by that with respect to entries of similar resolution.

The Analysed column shows the number of residues for which the backbone conformation was analysed, and the total number of residues.

Mol	Chain	Analysed	Favoured	Allowed	Outliers	Percentiles	
1	A	75/77 (97%)	74 (99%)	1 (1%)	0	100	100
2	B	70/72 (97%)	70 (100%)	0	0	100	100
All	All	145/149 (97%)	144 (99%)	1 (1%)	0	100	100

There are no Ramachandran outliers to report.

5.3.2 Protein sidechains (i)

In the following table, the Percentiles column shows the percent sidechain outliers of the chain as a percentile score with respect to all X-ray entries followed by that with respect to entries of similar resolution.

The Analysed column shows the number of residues for which the sidechain conformation was analysed, and the total number of residues.

Mol	Chain	Analysed	Rotameric	Outliers	Percentiles	
1	A	64/69 (93%)	63 (98%)	1 (2%)	62	78

Continued on next page...

PRELIMINARY VALIDATION REPORT

Continued from previous page...

Mol	Chain	Analysed	Rotameric	Outliers	Percentiles	
2	B	62/65 (95%)	61 (98%)	1 (2%)	62	78
All	All	126/134 (94%)	124 (98%)	2 (2%)	62	78

All (2) residues with a non-rotameric sidechain are listed below:

Mol	Chain	Res	Type
1	A	71	SER
2	B	3	SER

Some sidechains can be flipped to improve hydrogen bonding and reduce clashes. There are no such sidechains identified.

5.3.3 RNA (i)

Mol	Chain	Analysed	Backbone Outliers	Pucker Outliers
3	C	18/19 (94%)	0	0
3	D	18/19 (94%)	0	0
All	All	36/38 (94%)	0	0

There are no RNA backbone outliers to report.

There are no RNA pucker outliers to report.

5.4 Non-standard residues in protein, DNA, RNA chains (i)

There are no non-standard protein/DNA/RNA residues in this entry.

5.5 Carbohydrates (i)

There are no carbohydrates in this entry.

5.6 Ligand geometry (i)

6 ligands are modelled in this entry.

In the following table, the Counts columns list the number of bonds (or angles) for which Mogul statistics could be retrieved, the number of bonds (or angles) that are observed in the model and the number of bonds (or angles) that are defined in the Chemical Component Dictionary. The Link column lists molecule types, if any, to which the group is linked. The Z score for a bond

PRELIMINARY VALIDATION REPORT

length (or angle) is the number of standard deviations the observed value is removed from the expected value. A bond length (or angle) with $|Z| > 2$ is considered an outlier worth inspection. RMSZ is the root-mean-square of all Z scores of the bond lengths (or angles).

Mol	Type	Chain	Res	Link	Bond lengths			Bond angles		
					Counts	RMSZ	$\#\|Z\| > 2$	Counts	RMSZ	$\#\|Z\| > 2$
4	SO4	E	1	-	4,4,4	0.13	0	6,6,6	0.09	0
5	NO3	F	4	-	1,3,3	0.70	0	0,3,3	0.00	-
4	SO4	E	2	-	4,4,4	0.15	0	6,6,6	0.09	0
5	NO3	F	2	-	1,3,3	0.74	0	0,3,3	0.00	-
5	NO3	F	3	-	1,3,3	0.55	0	0,3,3	0.00	-
5	NO3	F	1	-	1,3,3	0.70	0	0,3,3	0.00	-

There are no bond length outliers.

There are no bond angle outliers.

There are no chirality outliers.

There are no torsion outliers.

There are no ring outliers.

No monomer is involved in short contacts.

5.7 Other polymers (i)

There are no such residues in this entry.

5.8 Polymer linkage issues (i)

There are no chain breaks in this entry.

PRELIMINARY VALIDATION REPORT

6 Fit of model and data (i)

6.1 Protein, DNA and RNA chains (i)

In the following table, the column labelled '#RSRZ> 2' contains the number (and percentage) of RSRZ outliers, followed by percent RSRZ outliers for the chain as percentile scores relative to all X-ray entries and entries of similar resolution. The OWAB column contains the minimum, median, 95th percentile and maximum values of the occupancy-weighted average B-factor per residue. The column labelled 'Q< 0.9' lists the number of (and percentage) of residues with an average occupancy less than 0.9.

Mol	Chain	Analysed	<RSRZ>	#RSRZ>2			OWAB(Å^2)	Q<0.9
1	A	77/77 (100%)	0.17	3 (3%)	39	46	46, 58, 101, 121	0
2	B	72/72 (100%)	0.16	3 (4%)	36	43	48, 57, 91, 109	0
3	C	19/19 (100%)	0.24	0	100	100	55, 67, 102, 107	0
3	D	19/19 (100%)	0.05	0	100	100	57, 70, 98, 103	0
All	All	187/187 (100%)	0.16	6 (3%)	47	54	46, 59, 98, 121	0

All (6) RSRZ outliers are listed below:

Mol	Chain	Res	Type	RSRZ
2	B	71	SER	3.0
1	A	-3	GLY	2.9
2	B	1	SER	2.6
1	A	25	ARG	2.4
1	A	26	ASP	2.3
2	B	2	ASP	2.0

6.2 Non-standard residues in protein, DNA, RNA chains (i)

There are no non-standard protein/DNA/RNA residues in this entry.

6.3 Carbohydrates (i)

There are no carbohydrates in this entry.

6.4 Ligands (i)

In the following table, the Atoms column lists the number of modelled atoms in the group and the number defined in the chemical component dictionary. The B-factors column lists the minimum,

PRELIMINARY VALIDATION REPORT

median, 95th percentile and maximum values of B factors of atoms in the group. The column labelled 'Q< 0.9' lists the number of atoms with occupancy less than 0.9.

Mol	Type	Chain	Res	Atoms	RSCC	RSR	B-factors($Å^2$)	Q<0.9
5	NO3	F	2	4/?	0.82	0.14	88,90,90,92	0
5	NO3	F	4	4/?	0.83	0.17	117,117,118,118	0
4	SO4	E	2	5/?	0.88	0.22	134,135,136,137	0
4	SO4	E	1	5/?	0.90	0.11	138,139,139,139	0
5	NO3	F	1	4/?	0.91	0.10	92,93,93,95	0
5	NO3	F	3	4/?	0.97	0.22	76,76,77,78	0

6.5 Other polymers (i)

There are no such residues in this entry.

PRELIMINARY VALIDATION REPORT
