# Supplementary material for: Structure and Sequence Determinants Governing the Interactions of RNAs with Influenza A Virus Non-Structural Protein NS1
Source: Viruses. 2020 Aug 27;12(9):947. doi: 10.3390/v12090947 (PMC7552008; doi:10.3390/v12090947)
Supplement: Supplementary file 1 [file viruses-12-00947-s001.zip › Supplementary_wacquiez_20200817.pdf]

viruses

MDPI

Supplementary Data

Structure and sequence determinants governing the interactions of RNAs with influenza A virus Non-Structural protein NS1

Alan Wacquiez[1,2,3], Franck Coste[3], Emmanuel Kut[1,2], Virginie Gaudon [3], Sascha Trapp[1,2], Bertrand Castaing[3]*, and Daniel Marc[1,2]*

[1] Equipe 3IMo, UMR1282 Infectiologie et Santé Publique, INRAE, Nouzilly, F-37380, France
[2] UMR1282 Infectiologie et Santé Publique, Université de Tours, Tours, F-37000, France
[3] Centre de Biophysique Moléculaire, UPR4301 CNRS, rue Charles Sadron, 45071 Orléans cedex

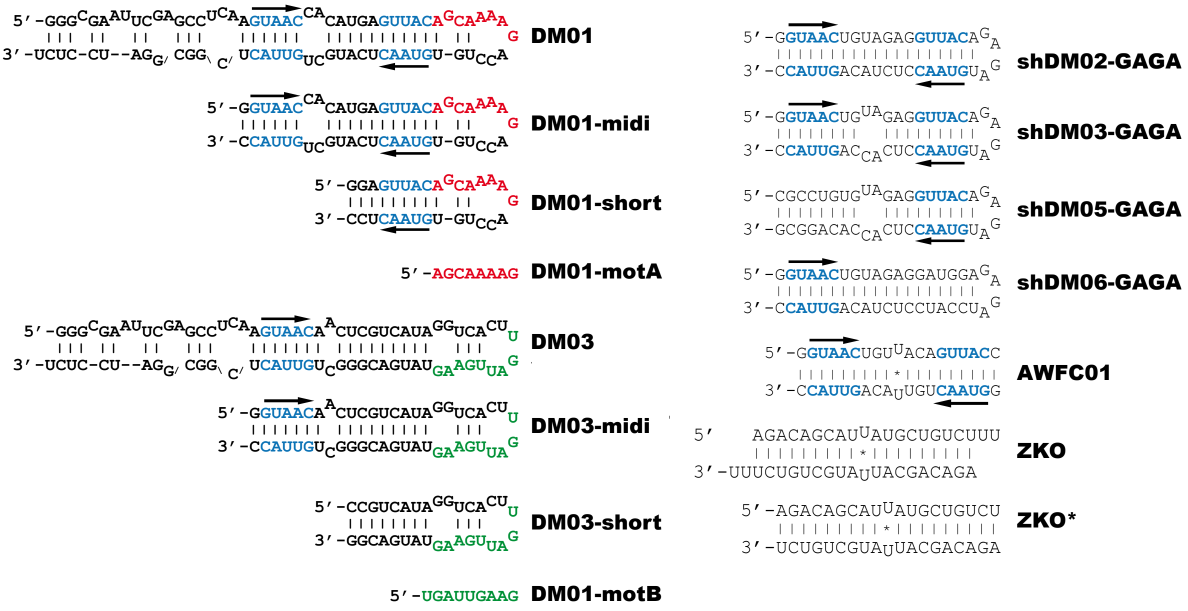

Figure S1: RNA probes used in this study

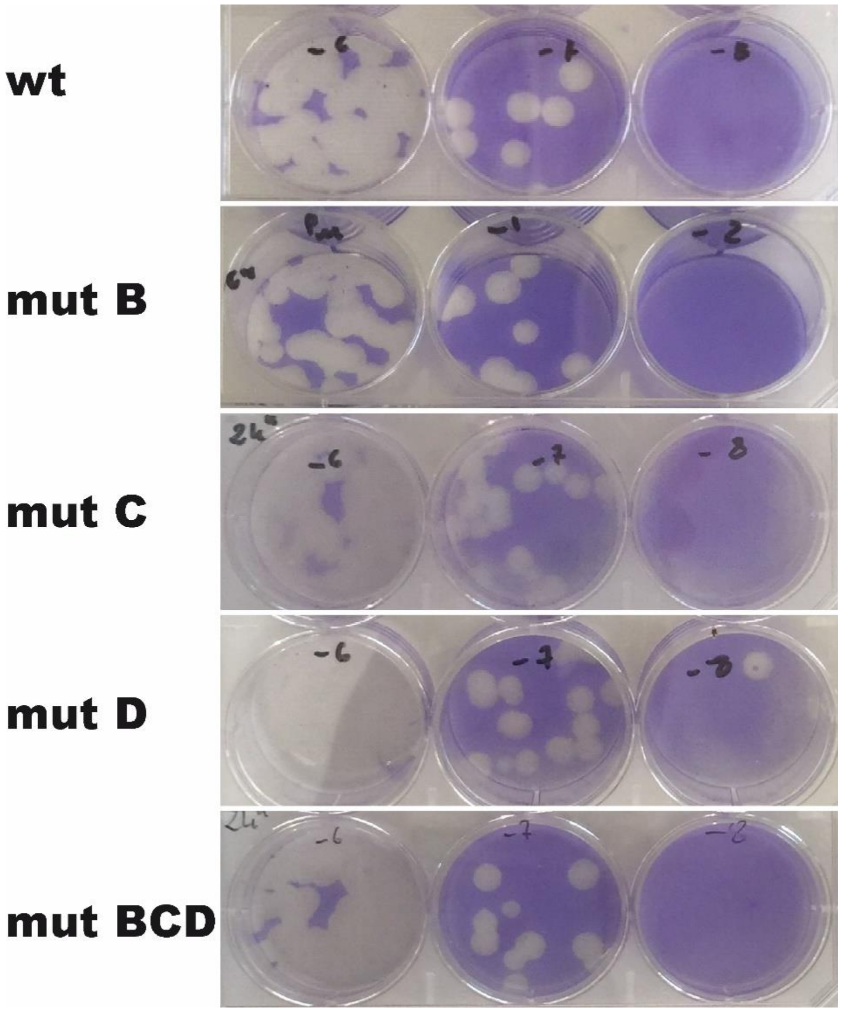

Figure S2: plaque phenotype of the mutant viruses. The viruses were titrated by plaque assays on MDCK cells, as described by Matrosovich et al., *Virol J* **2006**, *3*, 63.

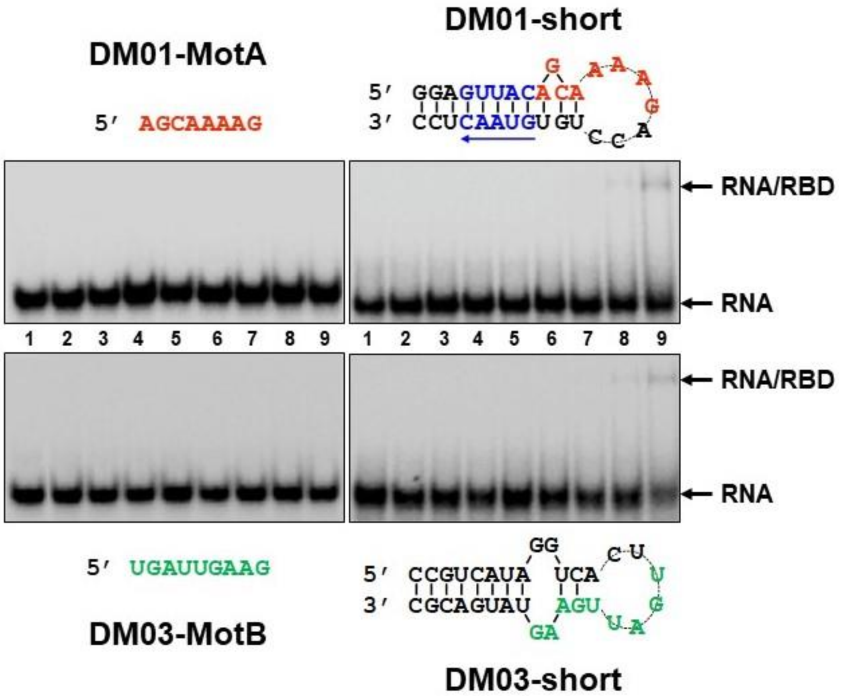

Figure S3. Comparative titration of DM01-short, DM01-MotA, DM03-short and DM03-MotB by RBD 0.1 nM of each 5′-[32P]-RNA probe was incubated alone (lane 1) or with 40, 80, 160, 320, 640, 1280, 2560 and 5120 nM of purified RBD of NS1 (H7N1) in standard conditions. The incubation mixtures were subsequently analyzed by EMSA as described in Materials and Methods section. Representative gel autoradiography are shown.

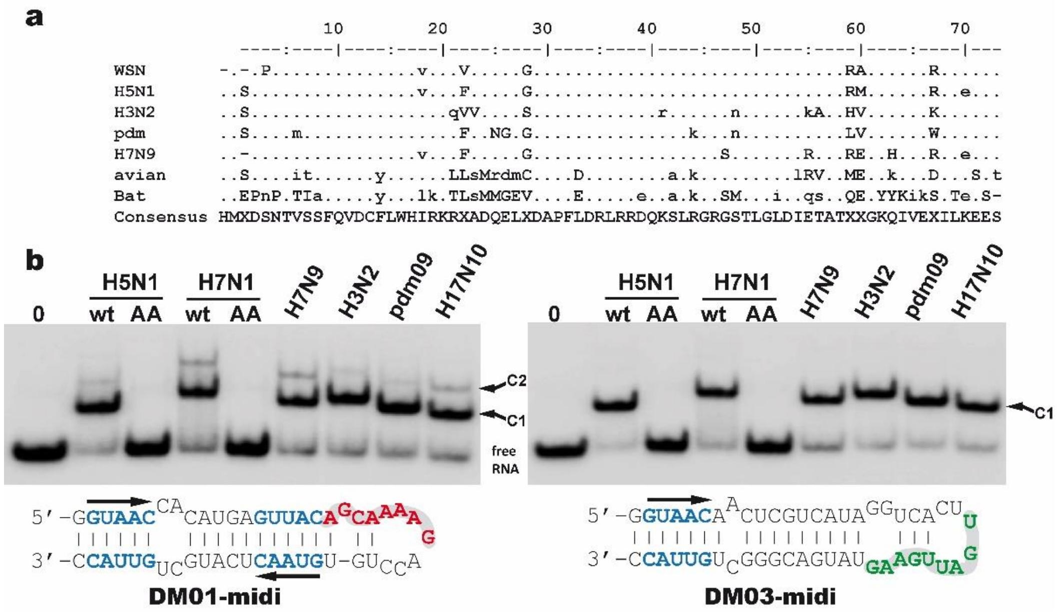

Figure S4. Comparative binding properties of selected RBDs with DM01-midi and DM03-midi (a) Alignment of the peptide sequences of the RBDs that were used. (b) The labeled RNAS were incubated with 20 nM of the indicated RBDs (AA indicates the R38A-K41A substitution). The incubation mixtures were subsequently analyzed by EMSA as described in Materials and Methods. Representative gel autoradiographies are shown.

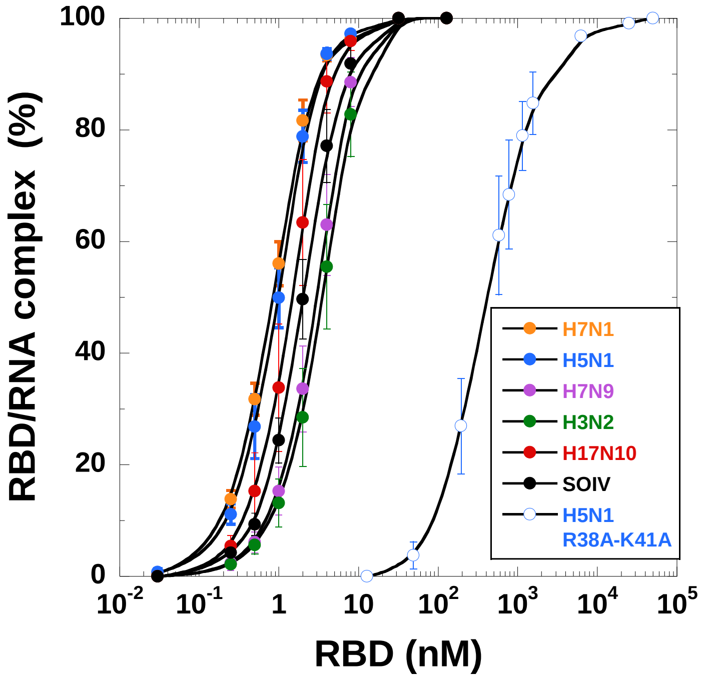

Figure S5. Titration curves obtained for the recognition of AWFC01 by various RBDs Radiolabeled AWFC01 was incubated in standard binding conditions with increasing concentrations of the NS1 RBD from various virus origins. The incubation mixtures were subsequently analyzed by EMSA, as illustrated in Figure 8. Dissociation constant (K_D) determined from titration experiments for each RBD were extracted from three dose-response curves. Under the conditions used, the RBD concentration needed for half-maximal RNA binding is very close to K_D. K_D values are shown in Table 2.

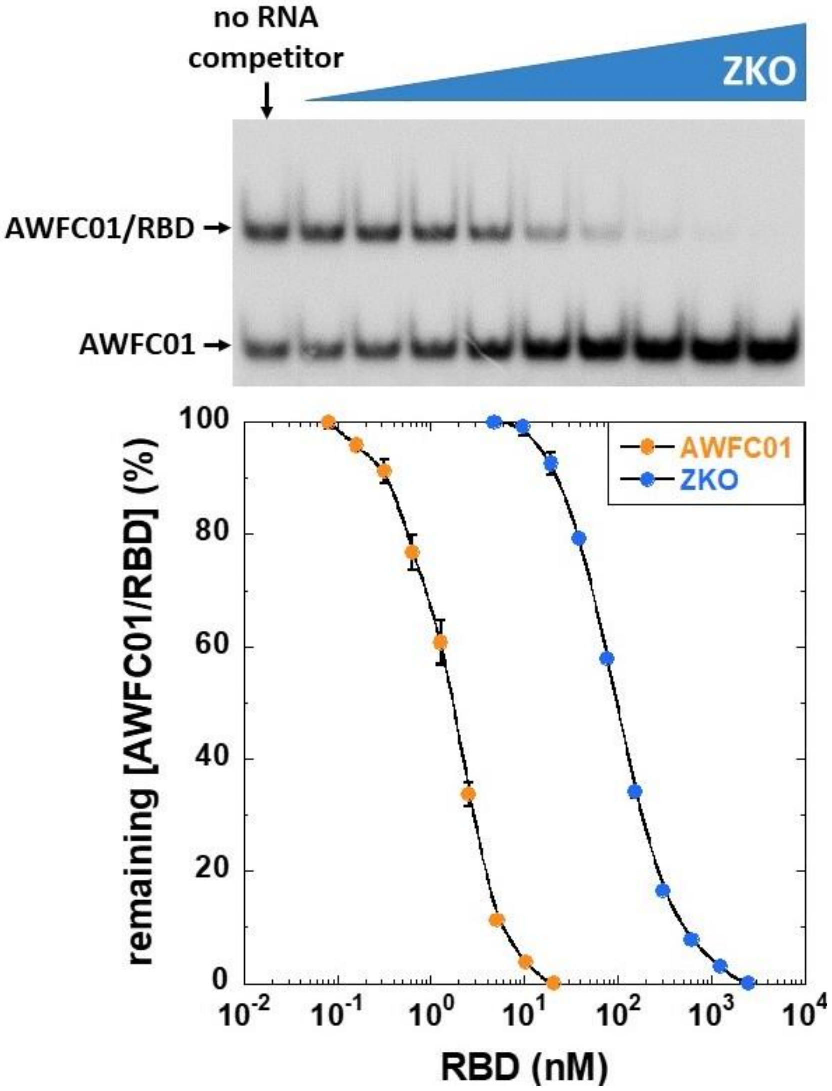

Figure S6. EC50app for the displacement of the preformed RBD/AWFC01 by other RNA probes Radiolabeled AWFC01 (0.1 nM) was pre-incubated (30 min. at 4°C) with 1 nM or 2 nM of NS1 RBD from H5N1 or H7N1, respectively. Subsequently, unlabeled competitors were added (0, 0.5, 1, 2.5, 3, 3.5, 4, 8 and 16 nM for AWFC01, DM01-midi, DM03-midi, shDM02-GAGA, shDM3-GAGA and shDM06-GAGA; or 0, 10, 20, 40, 80, 160, 320, 640, 1280 and 2560 nM of shDM05-GAGA and ZKO). After a further 30min-incubation, reaction mixtures were analyzed by EMSA in standard conditions. (a) Representative EMSA autoradiography of the dissociation of NS1 RBD from H5N1 bound to AWFC01 by increasing ZKO concentrations. (b) Dose response curves obtained with AWFC01 and ZKO as RNA competitors. The apparent half maximal effective concentration (EC50app) of RNA competitors which induces 50% dissociation of the preformed AWFC01 / RBD complex were extracted from these curves . EC50app was determined from at least three independent experiments and shown in Table 3.

Table S1. X-ray data collection and refinement statistics

**Data collection statistics**				
	**wtH7N1-RBD**	**wtH7N1-RBD / AWFC01**	**aaH7N1-RBD / AWFC01**	**wtH7N1-RBD / ZKO***
Radiation source	SOLEIL PROXIMA 2	SOLEIL PROXIMA 2	SOLEIL PROXIMA 2	SOLEIL PROXIMA 2
Wavelength (Å)	0.98009	0.98009	0.98009	0.98010
Spacegroup	$P3_121$	$P2_12_12_1$	$P2_12_12_1$	$P4_12_12$
cell dimensions: *a*, *b*, *c* (Å)	83.83, 83.83, 68.53	49.78, 60.40, 87.40	40.69, 102.04, 102.70	103.60, 103.60, 102.21
Resolution range (Å)	49.83-1.93 (1.97-1.93)	43.26-1.75 (1.78-1.75)	45.87-1.90 (1.94-1.90)	46.33-2.30 (2.38-2.30)
Total observations	239136 (9353)	177791 (10027)	179050 (11970)	336777 (34004)
Unique reflections	21170 (1030)	27291 (1481)	34557 (2202)	25358 (2431)
Completeness (%)	100.0 (100.0)	99.9 (100.0)	99.9 (100.0)	100.0 (100.0)
Multiplicity	11.3 (9.1)	6.5 (6.8)	5.2 (5.4)	13.3 (14.0)
R_{merge}^{a} (%)	7.8 (109.0)	4.9 (141.0)	4.3 (106.5)	8.6 (225.3)
Average $I/\sigma(I)$	15.9 (1.6)	16.7 (1.1)	16.3 (1.3)	18.8 (1.1)
$CC_{1/2}$ (%)	99.9 (70.4)	99.9 (57.1)	99.9 (48.1)	99.9 (55.3)
**Refinement and model statistics**				
Resolution range (Å)	49.83-1.93	43.26-1.75	37.80-1.90	46.33-2.30
Number of reflections used	21170	27229	34486	25302
R_{work} ^{b} / R_{free} ^{c} (%)	17.6/19.3	17.3/20.4	16.8/19.2	19.1/22.1
Average B values (Å^2)				
All atoms	54.36	42.07	50.05	67.29
Protein chain A atoms	51.50	39.10	39.74	63.15
Protein chain B atoms	56.67	39.54	43.45	61.32
RNA chain C atoms	-	43.28	60.93	73.73
RNA chain D atoms	-	43.77	63.08	73.90
Ethane-1,2-diol atoms	86.20	-	56.67	-
Polyethylene glycol atoms	66.33	63.74	-	-
Sulfate atoms	-	-	-	137.81
Nitrate atoms	-	-	-	94.87
Water atoms	51.16	45.56	49.77	58.57
Root mean square deviation from ideality				
Bond lengths (Å)	0.020	0.005	0.005	0.004
Bond angles (°)	1.500	0.818	0.778	0.667
Ramachandran analysis (% of residues)				
Favoured regions / Allowed regions / Outliers	99.3/0.7/0.0	100.0/0.0/0.0	100.0/0.0/0.0	99.3/0.7/0.0
Number of atoms				
Protein chain A	591	561	579	595
Protein chain B	587	572	569	570
RNA chain C	-	388	399	399
RNA chain D	-	391	399	399
Ethane-1,2-diol	12	-	36	-
Polyethylene glycol	14	67	-	-
Sulfate	-	-	-	10
Nitrate	-	-	-	16
Water	66	156	158	56
**PDB code**	**6SW8**	**6SX0**	**6SX2**	**6ZLC**

[a] $R_{merge} = \sum_h \sum_i |I_{h,i} - \langle I \rangle_h| / \sum_h \sum_i I_{h,i}$ where $<I>_h$ is the mean intensity of the symmetry-equivalent reflections.

[b] $R_{work} = \sum_h ||F_o| - |F_c|| / \sum_h |F_o|$, where F_o and F_c are the observed and calculated structure factor amplitudes, respectively, for reflection h.

[c] R_{free} is the *R* value for a subset of 5% of the reflection data, which were not included in the crystallographic refinement.

a

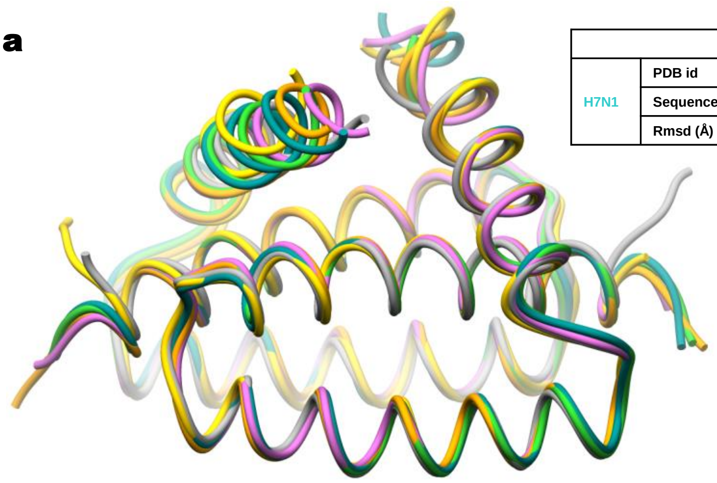

		H1N1	H3N2	H5N1	H17N10	H18N11
H7N1	PDB id	3M8A	1AIL	3F5T	5BXZ	5BY1
	Sequence Identity (%)	68	67	65	59	56
	Rmsd (Å)	0.63	0.47	1.05	1.42	2.18

b

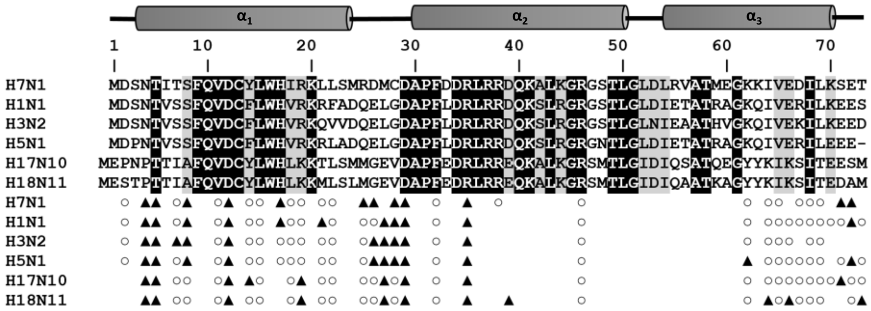

Figure S7. Comparison of H7N1 NS1 RBD with allele A RBDs (a) Superimposition of NS1 RBD 3D structures with corresponding root mean square deviation values (rmsd) and primary sequence identity. (b) Primary structure of NS1 RBD of known 3D structures (open circles = interfacing residues; black triangles = interfacing residues making inter-chain H-bonds).

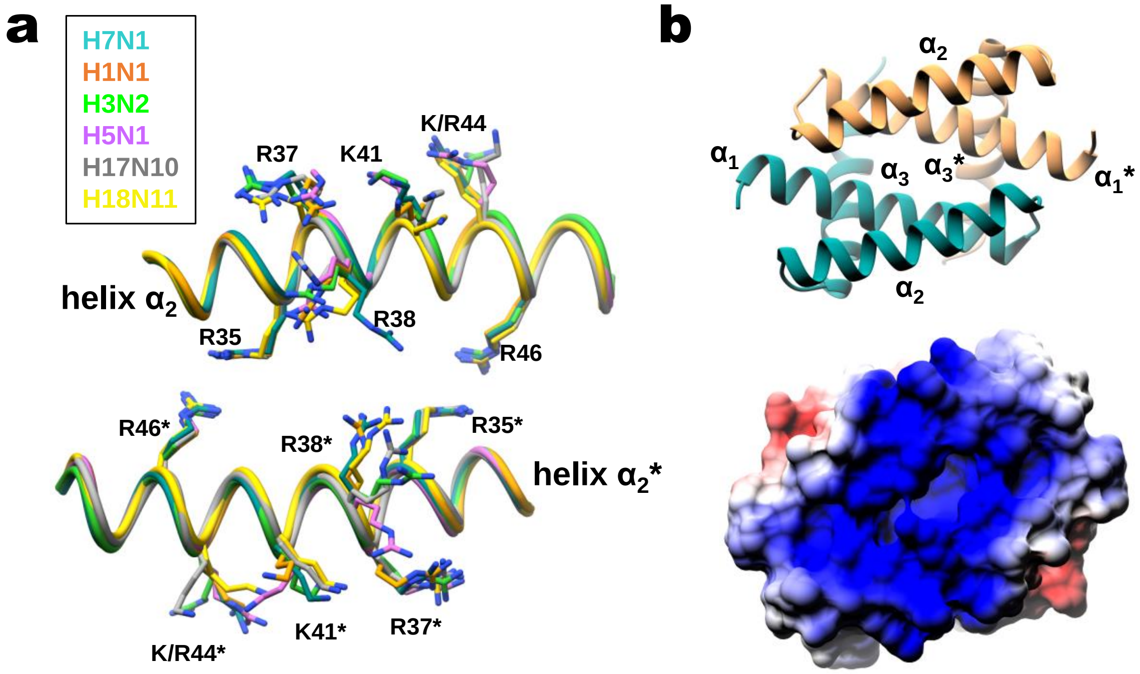

Figure S8. RNA binding interface of NS1 RBD (a) Conserved positively charged residues of H7N1 RBD α2 helix. (b) Electrostatic potential surface.

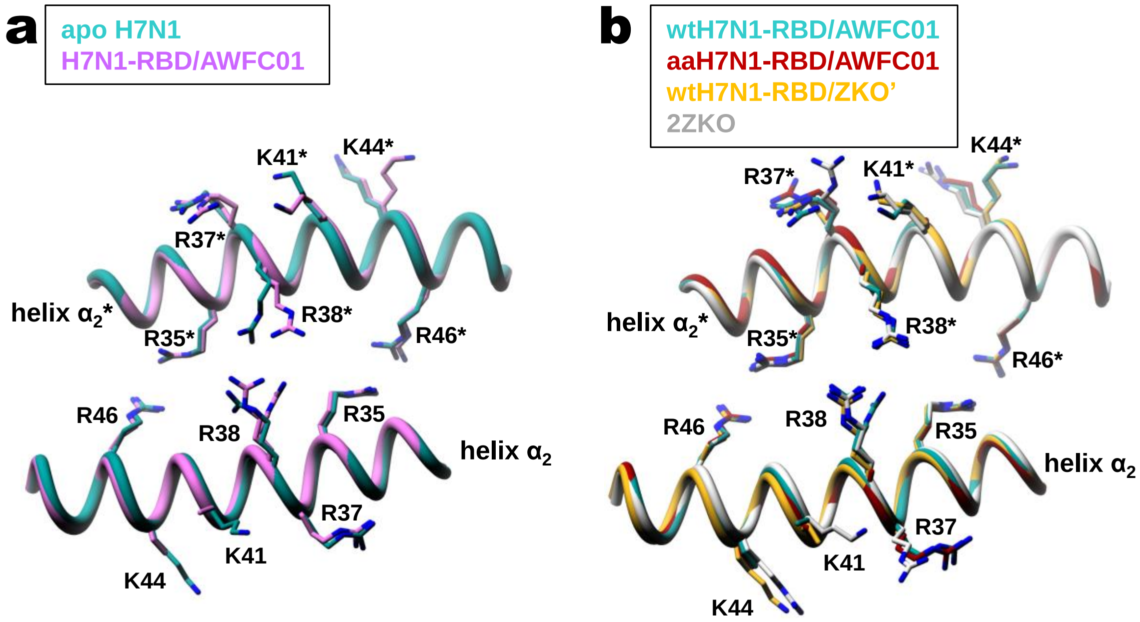

Figure S9. Conserved positively charged residues of the RNA binding interface (a) Superimposition of apo wtH7N1 RBD (dark cyan) and wtH7N1-RBD/AWFC01 complex (orchid). (b) Superimposition of wtH7N1-RBD/AWFC01 (dark cyan), aaH7N1-RBD/AWFC01 (dark red), wtH7N1-RBD/ZKO* (goldenrod) and 2ZKO (light gray) complexes.

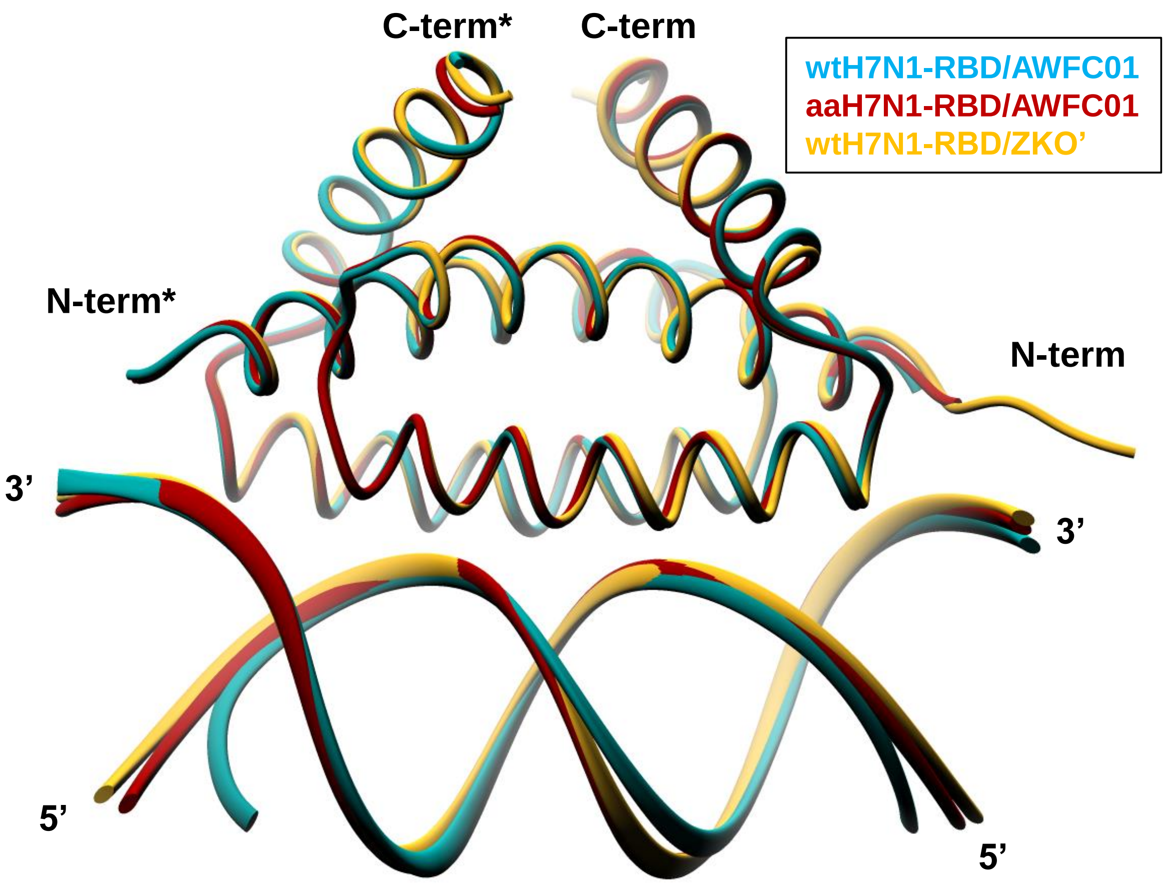

Figure S10. Comparison of the protein backbones in the structure of the three RBD-RNA complexes Superimposed are the alpha carbon backbones of the complexes wtH7N1-RBD/AWFC01 (dark cyan), aaH7N1-RBD/AWFC01 (dark red) and wtH7N1-RBD/ZKO* (goldenrod) complexes.

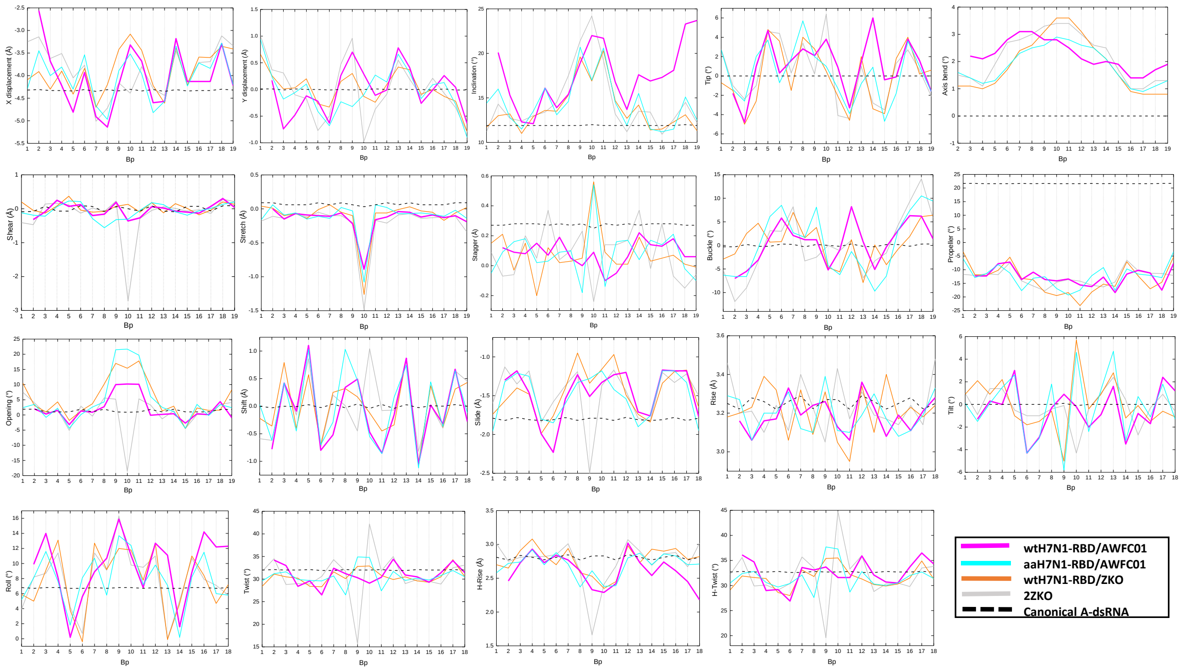

Bp 1 2 3 4 5 6 7 8 9 10 11 12 13 14 15 16 17 18 19

AWFC01 5'-G G U A A C U G U U A C A G U U A C C-3'
3'-C C A U U G A C A U U G U C A A U G G-5'

ZKO' 5'-A G A C A G C A U U A U G C U G U C U-3'
3'-U C U G U C G U A U U A C G A C A G A-5'

Figure S11. RNA structural parameters from Curves+ Plots of base pair-axis, intra-base pair parameters and inter-base pair parameters versus RNA base pair for wtH7N1-RBD/AWFC01 (pink), aaH7N1-RBD/AWFC01 (cyan), wtH7N1-RBD/ZKO (orange) and 2zko (grey) complexes.

a

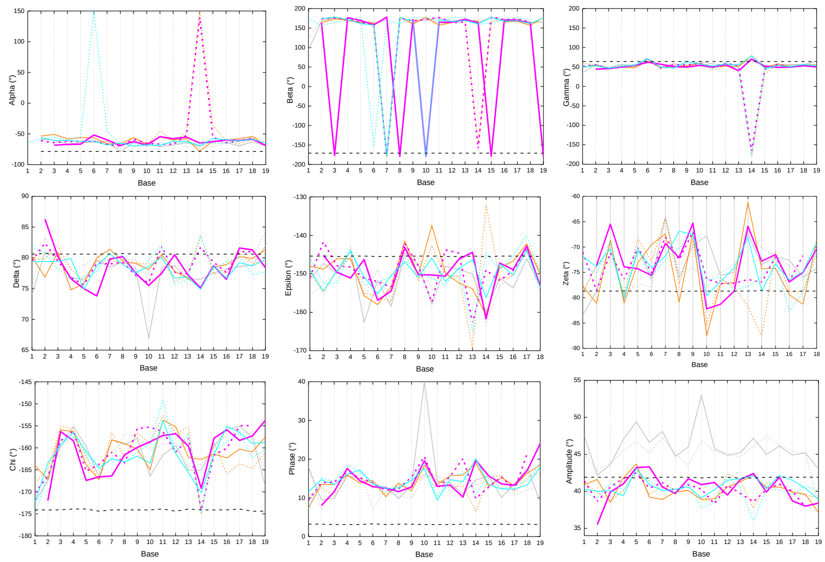

b

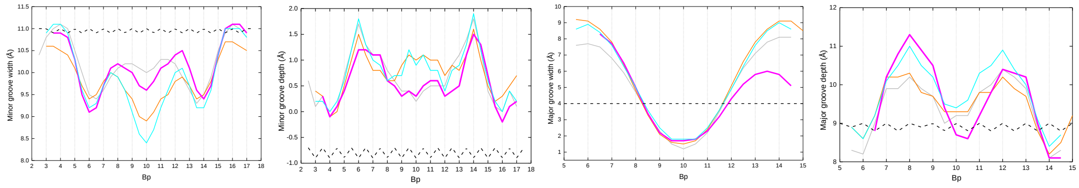

Figure S12. RNA structural parameters from Curves+ (continuation of Figure S10) (a) RNA backbone bond torsion angles. (b) RNA grooves width and depth.

© 2020 by the authors. Submitted for possible open access publication under the terms and conditions of the Creative Commons Attribution (CC BY) license (http://creativecommons.org/licenses/by/4.0/).
